# Efficacy and safety of ursodeoxycholic acid in children with cholestasis: A systematic review and meta-analysis

**DOI:** 10.1371/journal.pone.0280691

**Published:** 2023-01-31

**Authors:** Liang Huang, Siyu Li, Jingjing Chen, Yu Zhu, Ke Lan, Linan Zeng, Xuehua Jiang, Lingli Zhang

**Affiliations:** 1 West China School of Pharmacy, Sichuan University, Chengdu, China; 2 Department of Pharmacy, West China Second University Hospital, Sichuan University, Chengdu, China; 3 Evidence-based Pharmacy Center, West China Second University Hospital, Sichuan University, Chengdu, China; 4 NMPA Key Laboratory for Technical Research on Drug Products In Vitro and In Vivo Correlation, Chengdu, China; 5 Key Laboratory of Birth Defects and Related Diseases of Women and Children, Ministry of Education, Chengdu, China; 6 West China School of Medicine, Sichuan University, Chengdu, China; 7 Department of Pediatrics, West China Second University Hospital, Sichuan University, Chengdu, China; Zagazig University, EGYPT

## Abstract

**Objectives:**

Ursodeoxycholic acid (UDCA) is the main therapeutic drug for cholestasis, but its use in children is controversial. We conducted this study to evaluate the efficacy and safety of ursodeoxycholic acid in children with cholestasis.

**Methods:**

We searched Medline (Ovid), Embase (Ovid), Cochrane Central Register of Controlled Trials (CENTRAL), CNKI, WanFang Data and VIP from the establishment of databases to July 2022. Eligible studies included Chinese or English randomized controlled trials (RCTs) comparing the efficacy and safety of no UDCA (placebo or blank control) and UDCA in children with cholestasis. This study had been registered with PROSPERO (CRD42022354052).

**Results:**

A total of 32 RCTs proved eligible, which included 2153 patients. The results of meta-analysis showed that UDCA could improve symptoms of children with cholestasis (risk ratio 1.24, 95% CI 1.18 to 1.29; moderate quality of evidence), and serum levels of alanine aminotransferase, total bilirubin, direct bilirubin and total bile acid (low quality of evidence). For some children with specific cholestasis, UDCA could also effectively drop serum levels of aspartate aminotransferase (parenteral nutrition-associated cholestasis) and γ-glutamyl transferase (infantile hepatitis syndrome, parenteral nutrition-associated cholestasis). The most common adverse drug reactions (ADRs) of UDCA in children were gastrointestinal adverse reactions, with an incidence of 10.63% (67/630). There was no significant difference in the incidence of ADRs between UDCA and placebo/blank control groups (risk difference 0.03, 95%CI -0.01 to 0.06; moderate quality of evidence), and among children taking different UDCA doses (*P* = 0.27).

**Conclusion:**

The available short-term evidence showed that UDCA was effective and safe for children with cholestasis. Clinicians should use UDCA with caution (start with a low dose) until the long-term effect is further explored in future larger RCTs.

## Introduction

Cholestasis is a common pediatric disease with an incidence of about 1:5 000 to 1:2 500 [1], and being the top hepatobiliary disease leading to hospitalization in neonates and infants in China [2]. The main clinical symptoms of children with cholestasis include jaundice, pruritus, hepatosplenomegaly, and abnormal liver function [3, 4]. Usually, most children with cholestasis have a good prognosis if they are diagnosed and treated in time. However, if not detected and treated in time, it will seriously affect the growth and development of children, cause severe and irreversible neurological damage, and even lead to liver cirrhosis and death [5].

Ursodeoxycholic acid (UDCA), a commonly used hepatoprotective drug, has been the main treatment drug for cholestasis for many years [6, 7]. It is widely used to treat cholesterol-type gallstones [8] and cholestatic liver diseases (e.g., primary biliary cirrhosis) [9] in adults and most studies showed that UDCA was effective in improving symptoms of cholestasis in adults with good safety [10]. However, its off-label use in children is controversial. Some studies have found that UDCA is effective for cholestasis in children and children have good tolerance to it [11–14]. However, some researchers believed that UDCA was ineffective and unsafe in infants and neonates with cholestasis and might be associated with severe complications (cirrhosis, hepatocyte failure, and so forth.), disease progression and death, especially when taking higher doses (20–40 mg/kg) /d) [15–17].

Given that UDCA use in children is off-label and mostly based on experience and current evidence for guiding clinical decision-making is limited, our study aims to conduct a systematic review to assess the efficacy and safety of UDCA in children with cholestasis and provide evidence for the rational use of UDCA in pediatrics.

## Methods

This study had been registered with PROSPERO (CRD42022354052).

### Eligibility criteria

Trials were considered eligible if they were randomized clinical trials (RCTs); enrolled participants with cholestasis and age ≤18 years; compared UDCA with no UDCA (defined as placebo or blank control); provided information on any of the primary outcome effective rate (a composite outcome defined as children with cholestasis with significant improvement in clinical symptoms and biochemical indicators (TBIL, DBIL, ALT, etc.) as a percentage of children receiving same treatments) and secondary outcomes which included liver function indicators (TBIL, DBIL, TBA, ALT, AST, GGT) and adverse drug reactions (ADRs); and were published in the English or Chinese language. Duplicate publications, unavailable full text and studies that cannot convert original data into the data we need were excluded.

### Search strategy and data sources

A systematic search of Medline (Ovid), Embase (Ovid), Cochrane Central Register of Controlled Trials (CENTRAL), CNKI, WanFang Data and VIP was conducted from the earliest publication date available to July 20, 2022. The reference lists of included studies and reviews identified in the search were screened for additional studies. The search strategy was adjusted specifically for each database. It included a combination of medical subject headings and free text terms for ("child" or "kid" or "baby" or "pediatrics" or "neonate" or "newborn" or "infant" or "adolescent" or "teenager") and ("UDCA" or "ursodeoxycholic acid" or "ursodeoxycholic" or "Ursofalk" or "Ursolvan" or "Delursan" or "Ursodiol" or "Destolit" or "actigall" or "CholitUrsan"). We did not use any language restrictions during the search.

### Study selection

After removing duplicates, the titles and abstracts of the search results were screened for relevance by two authors (SL and JCh). Then, the full texts of preliminary included studies were further independently assessed for inclusion based on predetermined criteria. The final list of included studies was decided on by discussion between authors or judgment by a third reviewer (LH), with full agreement required before inclusion.

### Data extraction

Two authors (SL and JCh) completed data extraction and cross-check independently using piloted forms. The data extracted from each study included basic information of literature (title, the first author, publication year, and so forth.); baseline participant characteristics (sex, age, comorbidity, and so forth.); study design (sample size, inclusion criteria, study drug and control treatment (generic drug name, dosage form, administration route, dose, frequency and duration, and so forth.), follow-up duration, and so forth.); end-point data; key information for risk of bias assessment.

### Quality assessment

For the methodology quality assessment of RCTs, two authors (SL and JCh) independently use the Cochrane Collaboration risk of bias tool (Rob 2.0) to assess the risk of bias across five domains (randomization process, deviations from intended interventions, missing outcome data, blinding of outcome assessment, selection of the reported result).

For the quality of evidence for each outcome, two authors (SL and JCh) independently used the Grading of Recommendations, Assessment, Development and Evaluation (GRADE) quality assessment tool [18] to assess it. GRADE is a valid measure of the quality of evidence. Based on the comprehensive evaluation of included studies, rate each outcome as high, moderate, low, or very low quality of evidence. RCTs started at a high level. Downgrading was based on "limitations in study design and/or execution", "inconsistency of results", "indirectness of evidence", "imprecision of results" and "publication bias"; and upgrading was based on "large magnitude of effect", "confounders which may be working to reduce the demonstrated effect" and "dose-response gradient".

Discrepancies among the reviewers were resolved through discussions and consensus.

### Subgroup analysis

All outcomes except ADRs were analyzed for different cholestatic conditions (such as parenteral nutrition-associated cholestasis (PNAC), infantile hepatitis syndrome (IHS) and finally cytomegalovirus hepatitis). The primary outcome effective rate and ADRs were additionally analyzed for different UDCA doses. All subgroup analyses were pre-specified.

### Data synthesis

Meta-analysis was performed in RevMan 5.4. Continuous variables were presented as risk ratio (RR) or risk difference (RD), and for categorical variables, mean difference (MD) was used as effect size. Point estimates and 95% confidence intervals (95% CIs) were calculated for each effect size. The heterogeneity among included studies was tested by the Q-Test (Significance level α = 0.1), and the I^2^ indicator quantified the degree of heterogeneity. If I^2^ < 50% and *P*> 0.1, it meant that there was no heterogeneity or only low-to-moderate degree heterogeneity among the studies and the fixed effects model based on the Mantel and Haenszel method was used for meta-analysis; If I^2^≥ 50% or P<0.1, the source of heterogeneity was further analyzed. After excluding obvious clinical heterogeneity, the DerSimonian-Laird random effects method combined effect size. Pre-specified sensitivity analyses were performed by changing the statistical analysis model (fixed effect model/random effect model), excluding small sample studies (number of participants in any group ≤ 30) to assess the stability of the results. In addition, The asymmetry of funnel plots was used to identify publication bias.

## Results

### Study selection and study characteristics

**Fig 1:**
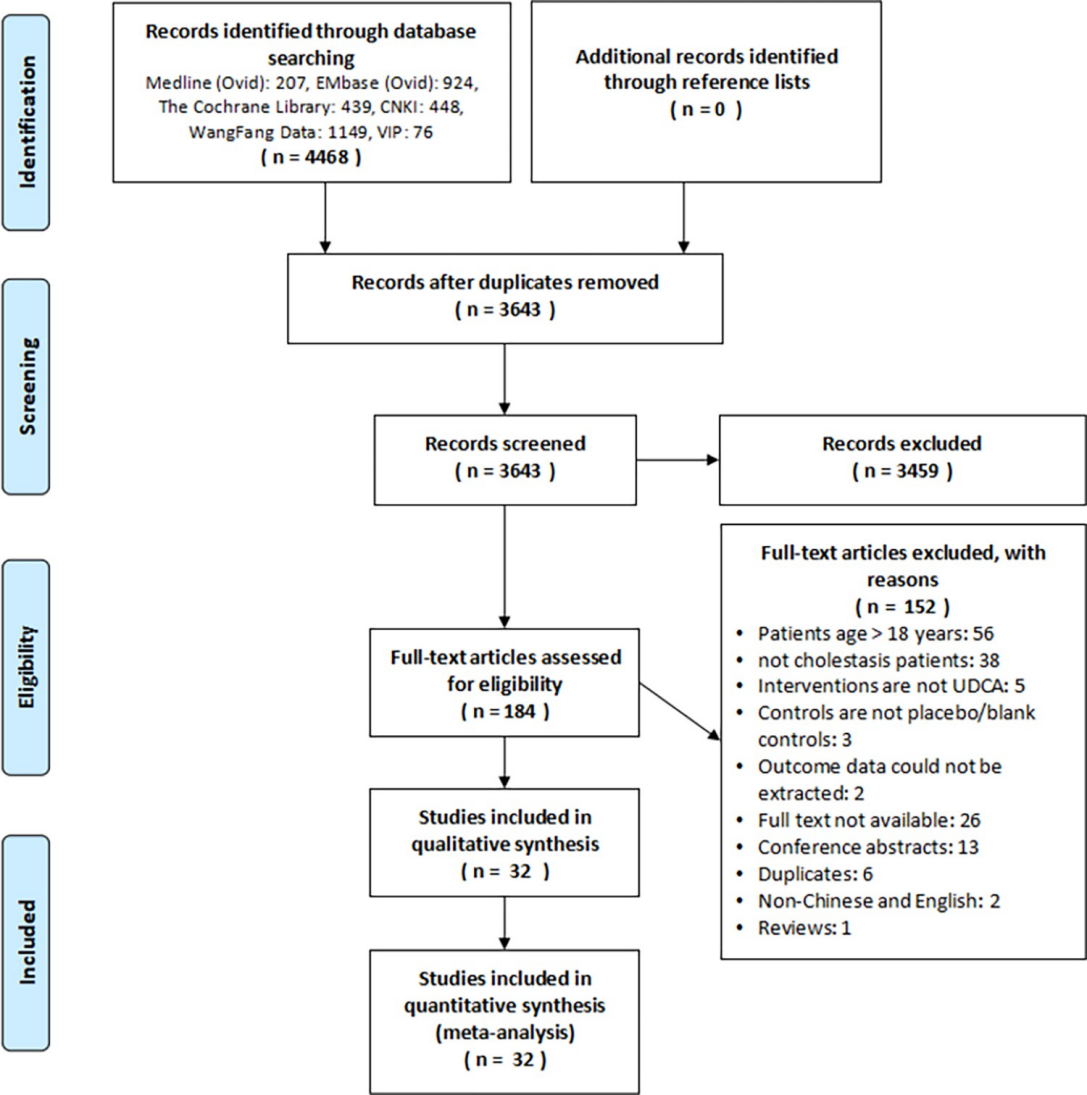
Study selection process and results.

The systematic search of studies published before July 20, 2022, identified 4468 records (Fig 1). Screening and full-text article analysis identified 32 eligible RCTs [2, 13, 19–48] (31 were conducted in China and one in Italy), including 2153 patients, of which 1086 (50.44%) received UDCA and 1067 (49.56%) did not (received placebo or blank control). These studies evaluated the effects of UDCA on IHS (n = 8), PNAC (n = 8), cytomegalovirus hepatitis (n = 6), and infantile cholestasis (cause unknown or not reported in articles) (n = 10). Moreover, the median (interquartile range) duration of follow-up was 14d (14d to 23d), and the median proportion of males was 55.5% (Table 1).

**Table 1:** Included studies and basic characteristics of patients.

Study ID	Country	Publication year	Study design	Sample size (T/C)	Age (d: day(s), w: week(s), m: month(s), y: year(s))	Sex (male/total)	Condition	Interventions	Medication regimen (Dosage, Frequency, Administration Route)	Controls	Duration of follow-up (days)	Outcomes
**Sertac Arslanoglu, 2007**	Italy	2007	RCT	15/14	NR	NR	Parenteral nutrition-associated cholestasis (PNAC)	UDCA	5~20 mg/kg/d, qid, injected via gastric tube	Placebo	42d	GGT
**Fan Jianping, 2008**	China	2008	RCT	29/25	48±21 d	30/54	Cytomegalovirus hepatitis	UDCA	10 mg/kg/d, tid, po	Blank control	14d	GGT TBIL DBIL TBA
**High Venus, 2009**	China	2009	RCT	45/40	2.35 m (25 d to 6 m)	44/85	Infantile hepatitis syndrome (IHS)	UDCA	15~25 mg/kg/d, bid /tid, po	Blank control	14d	Effective rate ALT GGT TBIL DBIL TBA ADRs
**Wang Yongbo, 2009**	China	2009	RCT	13/13	NR	14/26	Parenteral nutrition-associated cholestasis (PNAC)	UDCA	10 mg/kg/d, tid, po/injected via gastric tube	Blank control	30d	ALT GGT TBIL DBIL TBA
**Zhao Yang, 2012**	China	2012	RCT	42/40	48.6±10.2 d	52/82	Infantile hepatitis syndrome (IHS)	UDCA	20 mg/kg/d, bid, po	Blank control	14d	Effective rate ALT GGT TBIL DBIL TBA ADRs
**Li Changxiao, 2012**	China	2012	RCT	30/32	31 d-7 m	36/62	Infantile cholestasis	UDCA	10~15 mg/kg/d, bid, po	Blank control	NR	Effective rate ALT AST GGT TBIL DBIL ADRs
**Tang Qing, 2012**	China	2012	RCT	40/40	3.05±1.38 m	54/80	Infantile cholestatic hepatitis	UDCA	10~20 mg/kg/d, po	Blank control	14d	ALT GGT TBIL DBIL
**Wang Xiaoli, 2012**	China	2012	RCT	12/10	47±21 d	13/22	Cytomegalovirus hepatitis	UDCA	10 mg/kg/d, tid, po	Blank control	14d	ALT TBIL DBIL
**Wu Bixia, 2012**	China	2012	RCT	26/26	NR	27/52	Parenteral nutrition-associated cholestasis (PNAC)	UDCA	10 mg/kg/d, bid, po	Blank control	21d	Effective rate AST TBIL DBIL TBA ADRs
**Xu Hong, 2012**	China	2012	RCT	20/20	NR	23/40	Parenteral nutrition-associated cholestasis (PNAC)	UDCA	20~30 mg/kg/d, bid or tid, po/injected via gastric tube	Blank control	20d	ALT AST TBIL DBIL TBA
**Hu Jinping, 2013**	China	2013	RCT	32/33	35–107 d	35/65	Cytomegalovirus hepatitis	UDCA	10 mg/kg/d, bid, po	Blank control	28d	Effective rate ALT GGT TBIL DBIL TBA
**Li Shumin, 2013**	China	2013	RCT	20/20	NR	21/40	Parenteral nutrition-associated cholestasis (PNAC)	UDCA	10 mg/kg/d, tid, po/injected via gastric tube	Blank control	14d	ALT GGT TBIL DBIL TBA
**Wu Manpeng, 2013**	China	2013	RCT	20/15	NR	NR	Infantile hepatitis syndrome (IHS)	UDCA	10~20 mg/kg/d, bid, po	Blank control	NR	Effective rate ALT AST TBIL ADRs
**Ge Xingjing, 2014**	China	2014	RCT	20/20	33.5±15.7 d	22/40	Cytomegalovirus hepatitis	UDCA	10 mg/kg/d, bid, po	Blank control	14d	Effective rate ALT GGT TBIL DBIL TBA ADRs
**Beibei Sun, 2014**	China	2014	RCT	26/20	2.35 m (35 d to 6 m)	27/46	Infantile hepatitis syndrome (IHS)	UDCA	10~25 mg/kg/d, bid, po	Blank control	14d	Effective rate ALT GGT TBIL DBIL TBA ADRs
**Hu Huigang, 2015**	China	2015	RCT	30/30	39.3±17.2 d	33/60	Cytomegalovirus hepatitis	UDCA	10 mg/kg/d, bid, po	Blank control	28d	Effective rate ALT GGT TBIL DBIL TBA
**Gao Ming, 2016**	China	2016	RCT	43/43	49.1±10.2 d	60/86	Infantile hepatitis syndrome (IHS)	UDCA	10 mg/kg/d, bid, po	Blank control	21d	Effective rate ALT GGT TBIL DBIL TBA ADRs
**Parhati Ablimiti, 2016**	China	2016	RCT	30/30	3.98±0.34 m	38/60	Infantile hepatitis syndrome (IHS)	UDCA	10~20 mg/kg/d, bid, po	Blank control	7d	Effective rate ALT AST TBIL
**Qin Zhen, 2016**	China	2016	RCT	48/48	2.7±0.4 m	59/96	Cholestatic liver disease (ICH)	UDCA	20 mg/kg/d, NR,po	Blank control	30d	Effective rate ALT AST GGT TBIL DBIL ADRs
**Feng Lei, 2017**	China	2017	RCT	55/55	2.3±0.5 m	61/110	Infantile hepatitis syndrome (IHS)	UDCA	10 mg/kg/d, bid, po	Blank control	21d	Effective rate ALT GGT TBIL DBIL TBA
**Liu Yunfeng, 2017**	China	2017	RCT	32/32	NR	39/64	Parenteral nutrition-associated cholestasis (PNAC)	UDCA	30 mg/kg/d, tid, po	Blank control	14d	ALT AST TBIL ADRs
**Zhong Qiong, 2017**	China	2017	RCT	32/32	1 m to 6 m	39/72	Infantile cholestasis	UDCA	15 mg/kg/d, bid, po	Blank control	14d	Effective rate
**Zhou Li, 2017**	China	2017	RCT	40/40	30 d to 4 m	38/80	Cytomegalovirus hepatitis	UDCA	4~5 mg/kg/d, bid, po	Blank control	7d	Effective rate ALT GGT TBIL TBA
**Wang Wei, 2018**	China	2018	RCT	51/51	32.55±1.32 w	64/102	Parenteral nutrition-associated cholestasis (PNAC)	UDCA	10~20 mg/kg/d, NR, po	Blank control	14d	Effective rate ALT AST TBIL ADRs
**Yang Xingge, 2019**	China	2019	RCT	64/64	3.52±1.28 m	72/128	Cholestatic liver disease (ICH)	UDCA	20 mg/kg/d, qd, po	Blank control	14d	Effective rate ADRs
**Lin Guidi, 2019**	China	2019	RCT	35/35	10.21±0.71 d	33/70	Neonatal intrahepatic cholestasis	UDCA	20 mg/kg/d, NR, po	Blank control	14d	Effective rate ALT GGT TBIL DBIL ADRs
**Ma Qiushi, 2019**	China	2019	RCT	36/36	57±7 d	39/72	Neonatal cholestasis	UDCA	10~15 mg/kg/d, bid, po	Blank control	120d	Effective rate ALT AST GGT TBIL DBIL ADRs
**Xu Ya, 2019**	China	2019	RCT	43/43	1.21±0.52 y	52/86	Jaundice	UDCA	30 mg/kg/d, bid, po	Blank control	45d	Effective rate ALT TBIL TBA
**Yang Fengxia, 2019**	China	2019	RCT	44/47	68.38±15.92 d	50/91	Infantile hepatitis syndrome (IHS)	UDCA	50 mg/d, qd, po	Blank control	21d	Effective rate ALT GGT TBIL DBIL TBA ADRs
**Hu Haiyan, 2020**	China	2020	RCT	35/35	4.65±0.85 m	37/70	Infantile cholestasis	UDCA	10~15 mg/kg/d, NR, po	Blank control	14d	Effective rate ALT AST TBIL DBIL ADRs
**Wu Junfeng, 2021**	China	2021	RCT	44/44	64.97±15.95 d	40/88	Cholestatic liver disease (ICH)	UDCA	10~15 mg/kg/d, bid, po	Blank control	NR	Effective rate ALT GGT TBIL DBIL TBA ADRs
**Lan Jing, 2022**	China	2022	RCT	34/34	NR	50/68	Parenteral nutrition-associated cholestasis (PNAC)	UDCA	10 mg/kg/d, qd, po/injected via gastric tube	Blank control	NR	ALT AST GGT TBIL DBIL TBA ADRs

T: Treatment group; C: Control group; UDCA: Ursodeoxycholic acid; NR: Not reported; ALT: Alanine aminotransferase; AST: Aspartate aminotransferase; GGT: γ-glutamyl transferase; TBIL: Total bilirubin; DBIL: Direct bilirubin; TBA: Total bile acid

### Risk of bias assessment

None of the domains of any study was rated as having a high risk of bias. The overall risk of bias in studies was assessed as having some concerns, and bias in domains of deviations from intended interventions and outcome measurement was often a cause for concern. The results of each study’s risk of bias assessment and summary of the evidence quality assessment according to GRADE are in S1 Table.

### Primary analysis

The forest plots of the primary analyses for each outcome can be found in S1 Fig.

#### Primary outcome—Effective rate

**Fig 2:**
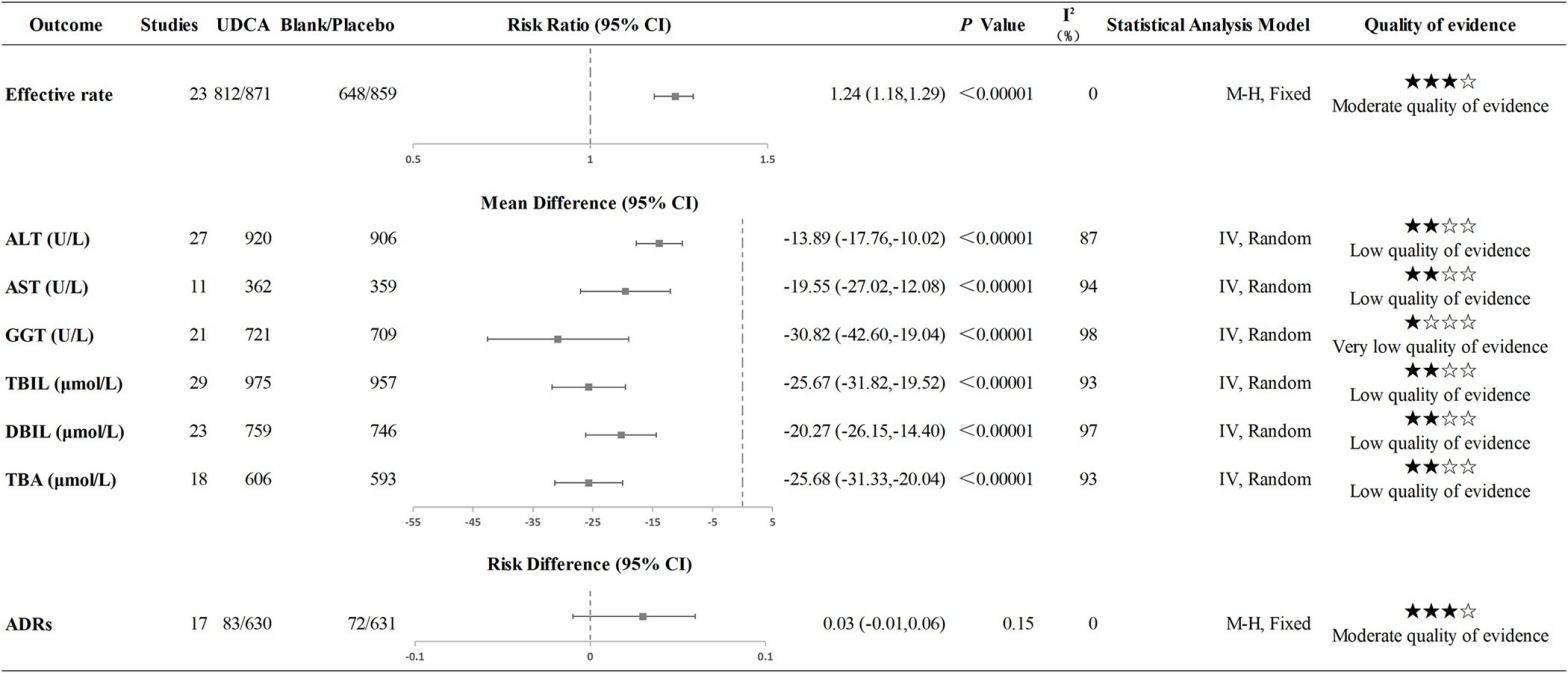
Results of primary meta-analyses.

A total of 23 studies [2, 13, 19–48] reported the effective rate of UDCA in children with cholestasis, with no statistical heterogeneity among studies (I^2^ = 0%, *P* = 0.95). The meta-analysis results showed that UDCA was more effective than placebo/blank control for children with cholestasis [RR = 1.24, 95%CI (1.18, 1.29), *P<*0.000001; Moderate quality of evidence] (Fig 2).

#### Liver function

*Total Bilirubin (TBIL)*. A total of 29 studies [2, 13, 20–30, 32–35, 37, 38, 44, 46–48] reported the effect of UDCA on TBIL and the meta-analysis results showed that UDCA could decrease the serum level of TBIL in children with cholestasis [MD = -25.67 μmol/L, 95%CI (-31,82, -19.52), *P*<0.000001; Low quality of evidence] (Fig 2).

*Direct Bilirubin (DBIL)*. Twenty-three studies [2, 13, 20–30, 32–35, 37, 38, 44, 46–48] reported the effect of UDCA on DBIL and the results of the meta-analysis showed that UDCA could decrease the serum level of DBIL in children with cholestasis [MD = -20.27 μmol/L, 95%CI (-26.15, -14.40), *P<*0.000001; Low quality of evidence] (Fig 2).

*Total Bile Acid (TBA)*. Eighteen studies [20–23, 27–30, 32–35, 38, 41, 45–48] reported the effect of UDCA on TBA and the results of the meta-analysis showed that UDCA could decrease the serum level of TBA in children with cholestasis [MD = -25.68 μmol/L, 95%CI (-31.33, -20.04), *P<*0.000001; Low quality of evidence] (Fig 2).

*Alanine Aminotransferase (ALT)*. A total of 27 studies [2, 13, 21–26, 28–39, 41, 42, 44–48] reported the effect of UDCA on ALT and the meta-analysis results showed that UDCA could decrease the serum level of ALT in children with cholestasis [MD = -13.89 U/L, 95%CI (-17.76, -10.02), *P*<0.000001; Low quality of evidence] (Fig 2).

*Aspartate Aminotransferase (AST)*. Eleven studies [2, 24, 27, 28, 31, 36, 37, 39, 42, 44, 48] reported the effect of UDCA on AST and the meta-analysis results showed that UDCA could decrease the serum level of AST in children with cholestasis [MD = -19.55 U/L, 95%CI (-27.02, -12.08), *P*<0.000001; Low quality of evidence] (Fig 2).

*Gamma-Glutamyl Transpeptidase (GGT)*. A total of 21 studies [13, 19–25, 29, 30, 32–35, 37, 38, 41, 44, 46–48] reported the effect of UDCA on GGT. The results of meta-analysis showed that UDCA could decrease the serum level of GGT in children with cholestasis [MD = -30.82 U/L, 95%CI(-42.60, -19,04), *P*<0.000001; Very low quality of evidence] (Fig 2).

#### ADRs

Seventeen studies [2, 13, 23, 24, 27, 31–33, 35, 37, 39, 42–44, 46–48] reported the incidence of ADRs of UDCA in children with cholestasis. Among the 630 children taking UDCA, 83 had ADRs, and the incidence of ADRs was 13.17%. Gastrointestinal ADRs, such as constipation or diarrhea (number of events, n = 56), loss of appetite (n = 6), vomiting (n = 3), and nausea (n = 2), were the most common, with an incidence of 10.63% (67/630). Rash (2.22%, 14/630), itching (0.48%, 3/630) and fever (0.16%, 1/630) also occurred occasionally after medication. The meta-analysis results showed no significant difference in ADR incidence between children taking UDCA and placebo/blank control [RD = 0.03, 95%CI (-0.01, 0.06), *P* = 0.15; Moderate quality of evidence] (Fig 2).

### Subgroup analysis

#### Primary outcome—Effective rate

**Fig 3:**
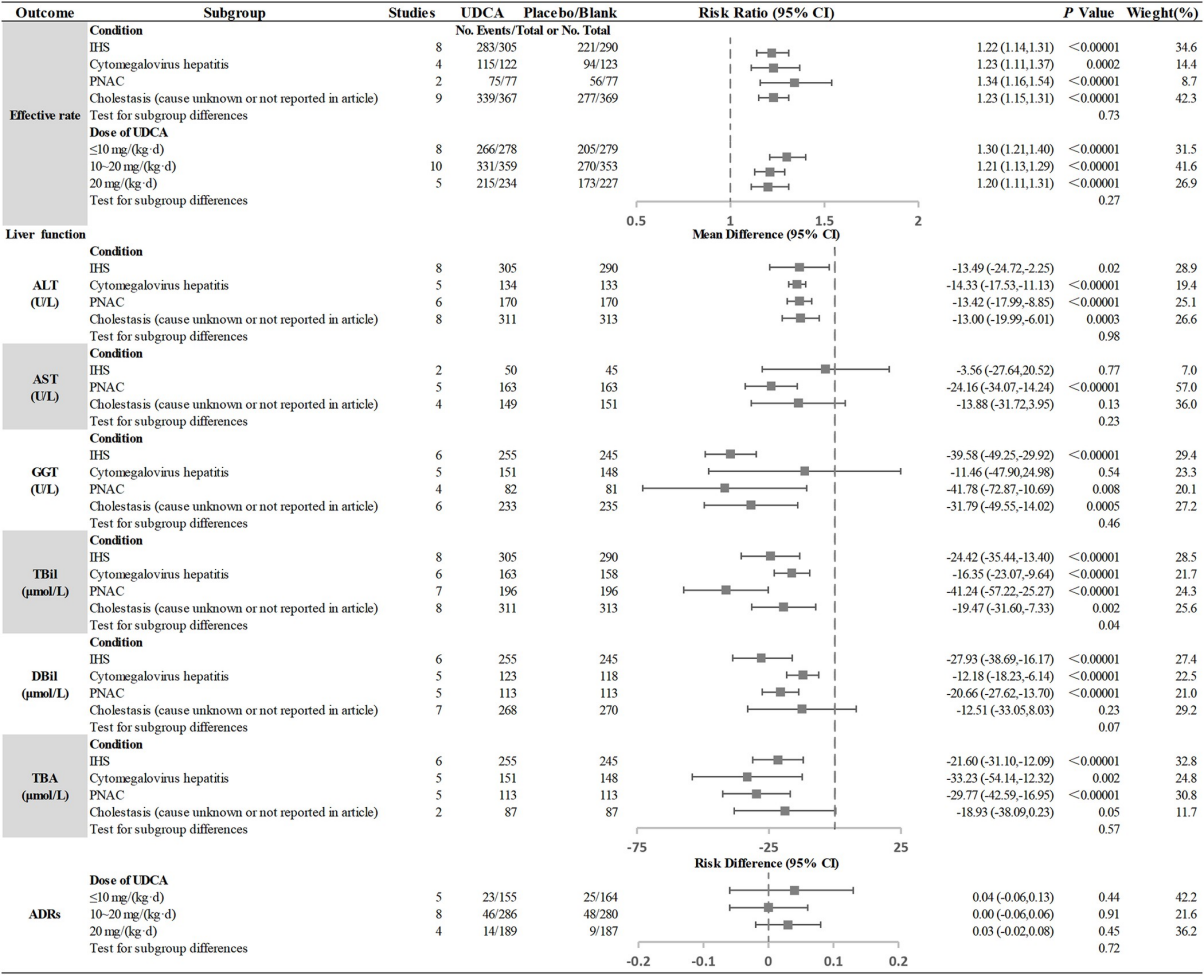
Results of subgroup analyses of outcomes.

We conducted the subgroup analyses of the effective rate of UDCA in children with cholestasis by different conditions and UDCA doses. The results showed that UDCA was more effective in IHS, cytomegalovirus hepatitis and PNAC than placebo/blank control, and there was no significant difference in efficacy for cholestasis caused by different conditions [RR = 1.22, 95%CI (1.14, 1.31); RR = 1.23, 95%CI (1.11, 1.37); RR = 1.34, 95%CI (1.16, 1.54); *P* = 0.73]. Moreover, there also was no significant difference in efficacy of different UDCA doses [UDCA≤10mg/kg/d: RR = 1.30, 95% CI (1.21, 1.40); 10~20mg/kg/d: RR = 1.21, 95% CI (1.13, 1.29); 20mg/kg/d: RR = 1.20, 95% CI (1.11, 1.31); *P* = 0.27] (Fig 3).

#### Liver function

*TBIL*. The results of subgroup analysis for TBIL showed that UDCA could decrease the elevated serum TBIL level in children with IHS, cytomegalovirus hepatitis and PNAC, and the effect of reducing TBIL was better than that of the placebo/blank control. In addition, there was a significant difference in the reduction extent in children with cholestasis caused by different conditions, and its effect was the best for PNAC, followed by IHS and finally cytomegalovirus hepatitis. [MD = -41.24 μmol/L, 95%CI (-57.22, -25.27); MD = -24.42 μmol/L, 95%CI (-35.44, -13.40); MD = -16.35 μmol/L, 95%CI (-23.07, -9.64); *P* = 0.04] (Fig 3).

*DBIL*. The results of subgroup analysis for DBIL showed that UDCA could reduce the rising serum DBIL in children with IHS, cytomegalovirus hepatitis and PNAC, and its effect was better than that of placebo/blank control. Moreover, there was no significant difference in children with cholestasis caused by different conditions [MD = -27.93 μmol/L, 95%CI (-38.69, -16.17); MD = -12.18 μmol/L, 95%CI (-18.23,-6.14); MD = -20.66 μmol/L, 95%CI (-27.62, -13.70); *P* = 0.07] (Fig 3).

*TBA*. The results of subgroup analysis for TBA showed that UDCA could reduce the rising serum TBA in children with IHS, cytomegalovirus hepatitis and PNAC, and its effect was better than that of placebo/blank control. In addition, there was no significant difference in children with cholestasis caused by different conditions [MD = -21.60 μmol/L, 95%CI (-31.10, -12.09); MD = -33.23 μmol/L, 95%CI (- 54.14, -12.32); MD = -29.77 μmol/L, 95%CI (-42.59, -16.95); *P* = 0.57] (Fig 3).

*ALT*. The subgroup analysis results for ALT showed that UDCA could reduce the rising serum ALT in children with IHS, cytomegalovirus hepatitis and PNAC, and its effect was better than that of placebo/blank control. And there was no significant difference in children with cholestasis caused by different conditions [MD = -13.49 U/L, 95%CI (-24.72, -2.25); MD = -14.33 U/L, 95%CI (-17.53, -11.13); MD = -13.42 U/L, 95%CI (-17.99, -8.85); *P* = 0.98] (Fig 3).

*AST*. The subgroup analysis for AST found that UDCA could decrease the elevated serum AST level in children with PNAC, and its AST reduction effect was better than that of placebo/blank control [MD = -24.16 U/L, 95%CI (-34.07, -14.24), *P*<0.00001]. However, there was no significant difference in AST between UDCA and placebo/blank control groups of children with IHS and cholestasis caused by other conditions [MD = -3.56 U/L, 95%CI (-27.64, 20.52); MD = -13.88 U/L, 95%CI (-31.72, 3.95)] (Fig 3).

*GGT*. The subgroup analysis for GGT showed that UDCA could reduce the rising serum GGT in children with IHS and PNAC, and its effect on reducing GGT was better than that of placebo/blank control [MD = -39.58 U/L, 95%CI (-49.25, -29.92), *P*<0.00001; MD = -41.78 U/L, 95%CI (-72.87, -10.69), *P* = 0.008]. However, there was no significant difference between UDCA and placebo/blank control in children with cytomegalovirus hepatitis [MD = -11.46 U/L, 95%CI (-47.90, 24.98)] (Fig 3).

#### ADRs

The subgroup analysis for ADRs found that the ADRs in the UDCA groups were similar to that in the placebo/blank control groups, and there was no significant difference among different UDCA doses. [UDCA≤10mg/kg/d: RD = 0.04, 95% CI (-0.06, 0.13); 10~20mg/kg/d: RD = 0.00, 95% CI (-0.06, 0.06); 20mg /kg/d: RD = 0.03, 95% CI (-0.02, 0.08); *P* = 0.27] (Fig 3).

### Sensitivity analysis

After changing the statistic model for combining effect sizes or excluding small sample studies, the directions of effect sizes of outcomes were unchanged. (See S2 Fig., which demonstrates the results of sensitivity analyses.)

### Publication bias

The funnel plots for each outcome can be found in S3 Fig. In funnel plots, except GGT, the scatter points representing each study were symmetrically distributed on both sides of the funnel plots, suggesting that there was no publication bias.

## Discussion

Our study results showed that UDCA could improve the clinical symptoms of children with cholestasis [RR = 1.24, 95%CI (1.18, 1.29), *P*<0.000001], and decrease serum levels of ALT, TBIL, DBIL and TBA. Moreover, for the rising serum TBIL caused by PNAC, UDCA had the best effect, followed by HIS and, finally, by cytomegalovirus hepatitis. For some children with specific cholestasis, UDCA could also effectively decrease serum levels of AST (PNAC) and GGT (IHS, PNAC).

Previous studies [9, 49] found that compared with a placebo, UDCA improved symptoms and serum levels of aminotransferases and bilirubin in adults with cholestasis. However, they had no significant benefit on long-term outcomes such as all-cause mortality, liver transplantation and varicose veins. In addition, a long-term, double-blind, randomized controlled trial found that the risk of developing cirrhosis, varicose veins, bile duct cancer, liver transplantation and death was twice in patients receiving high-dose UDCA (28 to 30 mg/kg/d) compared with patients receiving the placebo [50, 51]. There was no long-term, double-blind, randomized controlled trial conducted to evaluate the effect of UDCA on the long-term prognosis of children with cholestasis, but a retrospective study has shown that UDCA (15 to 40 mg/kg/d) had no benefit to neonates and infants with cholestasis (obstructive and non-obstructive) and put them at a higher risk of treatment failure [15, 16]. And it was associated with disease progression, severe complications (hepatocyte failure, ascites, vanishing bile duct syndrome, and so forth.) and death in children. Compared with cholestatic neonates and infants not taking UDCA, infants who received UDCA (15 to 30 mg/kg/d) had more than double the risk of hepatocyte failure and death [15]. Given that high doses of UDCA (15 to 40 mg/kg/d) may be associated with adverse clinical outcomes such as liver cirrhosis, hepatocyte failure and death. And the subgroup analyses results of this study showed that there was no significant difference in the efficacy in children taking different doses of UDCA [UDCA≤10mg/kg/d: RR = 1.30, 95% CI (1.21, 1.40); 10~20mg/kg/d: RR = 1.21, 95% CI (1.13, 1.29); 20mg/kg/d: RR = 1.20, 95% CI (1.11, 1.31); P = 0.27], low doses (10 mg/kg/d) UDCA may be more appropriate when used initially in children. Some studies showed that initial high doses could be considered reasonable in some children with diagnosed progressive familial intrahepatic cholestasis (the patients with high GGT-PFIC or proven PFIC3) [52, 53]. In addition, for obstructive cholestasis, surgical intervention is often chosen and UDCA is not recommended [54, 55].

Our study results showed that gastrointestinal ADRs were the most common in children receiving UDCA, with an incidence of 10.63% (67/630). There was no significant difference in the incidence of ADRs between UDCA and placebo/blank control and among children taking different doses of UDCA. Previous studies have shown similar findings in adults [9]—UDAC was not significantly different from placebo or no intervention in the risk of serious and non-serious adverse events.

### Limitations

This study had some limitations: First, our study only included Chinese and English literature, which may cause language bias. Second, the sample size of included studies was small (22 to 128 patients), and the duration of follow-up was short (7 days to 4 months), which could make it difficult to discover long-term or rare ADRs of UDCA. Third, all identified outcomes were short-term and intermediate outcomes, such as serum levels of transaminases and bilirubin, and lacking long-term and end-point outcomes, such as liver cirrhosis, varicose veins, liver transplantation, death, and so forth. It is unclear how effective UDCA improves long-term prognosis in children with cholestasis. Moreover, because the quality of included studies is not high and the inconsistency of results among studies, the quality of evidence is not high (very low to moderate quality of evidence). Our study results need to be confirmed by high-quality randomized controlled studies with larger samples and longer follow-up times. Finally, all the studies except one originate from China which could make it difficult to generalize the results.

## Conclusion

The available short-term evidence showed that UDCA was effective and safe for children with cholestasis, but clinicians still should be cautious and start with a low dose (10 mg/kg/d) when using it in children. Moreover, high-quality randomized controlled studies with larger samples and longer follow-up times to evaluate the long-term safety and efficacy of UDCA for children are limited, and this could become the direction of the following studies.

## Supporting information

S1 Table Results of the risk of bias assessment and summary of assessment of evidence quality according to GRADE. (DOCX)

S1 Fig Meta-analysis forest plots of the primary analyses. (PDF)

S2 Fig Results of sensitivity analyses. (PDF)

S3 Fig Funnel plots. (PDF)

## Abbreviations

UDCA: Ursodeoxycholic acid
ADEs: Adverse drug events
ADRs: Adverse drug reactions
PNAC: Parenteral nutrition-associated cholestasis
IHS: Infantile hepatitis syndrome
ALT: Alanine aminotransferase
AST: Aspartate aminotransferase
GGT: γ-glutamyl transferase
TBIL: Total bilirubin
DBIL: Direct bilirubin
TBA: Total bile acid
