# Supplementary material for: Efficacy and safety of ursodeoxycholic acid in children with cholestasis: A systematic review and meta-analysis
Source: PLoS One. 2023 Jan 31;18(1):e0280691. doi: 10.1371/journal.pone.0280691 (PMC9888709; doi:10.1371/journal.pone.0280691)
Supplement: S1 Table — (DOCX) [file pone.0280691.s001.docx]

**Table 1.** Risk of bias assessment results

| **Study ID** | **Bias arising from the randomisation process** | **Bias due to deviations from intended interventions** | **Bias due to missing outcome data** | **Blinding of outcome assessment** | **Bias in selection of the reported result** | **Overall risk of bias** |
| --- | --- | --- | --- | --- | --- | --- |
| Sertac Arslanoglu, 2007 | Low | Low | Low | Some concerns | Some concerns | Some concerns |
| Fan Jianping, 2008 | Low | Some concerns | Low | Low | Low | Some concerns |
| High Venus, 2009 | Low | Some concerns | Low | Some concerns | Low | Some concerns |
| Wang Yongbo, 2009 | Low | Some concerns | Low | Low | Low | Some concerns |
| Li Changxiao, 2012 | Low | Some concerns | Low | Some concerns | Low | Some concerns |
| Tang Qing, 2012 | Low | Some concerns | Low | Low | Low | Some concerns |
| Wang Xiaoli, 2012 | Low | Some concerns | Low | Low | Low | Some concerns |
| Wu Bixia, 2012 | Low | Some concerns | Low | Some concerns | Low | Some concerns |
| Xu Hong, 2012 | Low | Some concerns | Low | Low | Low | Some concerns |
| Zhao Yang, 2012 | Low | Some concerns | Low | Some concerns | Low | Some concerns |
| Hu Jinping, 2013 | Low | Low | Low | Some concerns | Low | Some concerns |
| Li Shumin, 2013 | Low | Some concerns | Low | Low | Low | Some concerns |
| Wu Manpeng, 2013 | Low | Some concerns | Low | Some concerns | Low | Some concerns |
| Ge Xingjing, 2014 | Low | Some concerns | Low | Some concerns | Low | Some concerns |
| Beibei Sun, 2014 | Low | Some concerns | Low | Some concerns | Low | Some concerns |
| Hu Huigang, 2015 | Low | Some concerns | Low | Some concerns | Low | Some concerns |
| Gao Ming, 2016 | Low | Some concerns | Low | Some concerns | Low | Some concerns |
| Parhatti Ablimiti, 2016 | Low | Some concerns | Low | Some concerns | Low | Some concerns |
| Qin Zhen, 2016 | Low | Some concerns | Low | Some concerns | Low | Some concerns |
| Feng Lei, 2017 | Low | Some concerns | Low | Some concerns | Low | Some concerns |
| Liu Yunfeng, 2017 | Low | Some concerns | Low | Low | Low | Some concerns |
| Zhong Qiong, 2017 | Low | Some concerns | Low | Some concerns | Low | Some concerns |
| Zhou Li, 2017 | Low | Some concerns | Low | Some concerns | Low | Some concerns |
| Wang Wei, 2018 | Low | Some concerns | Low | Some concerns | Low | Some concerns |
| Lin Guidi, 2019 | Low | Some concerns | Low | Low | Low | Some concerns |
| Ma Qiushi, 2019 | Low | Some concerns | Low | Some concerns | Low | Some concerns |
| Xu Ya, 2019 | Low | Some concerns | Low | Some concerns | Low | Some concerns |
| Yang Fengxia, 2019 | Low | Some concerns | Low | Some concerns | Low | Some concerns |
| Yang Xingge, 2019 | Low | Some concerns | Low | Some concerns | Low | Some concerns |
| Hu Haiyan, 2020 | Low | Some concerns | Low | Some concerns | Low | Some concerns |
| Wu Junfeng, 2021 | Low | Some concerns | Low | Some concerns | Low | Some concerns |
| Lan Jing, 2022 | Low | Some concerns | Low | Low | Low | Some concerns |

**Table 2.** Summary of assessment of evidence quality according to GRADE.

| **Outcome** | **Studies** | **UDCA** | **Blank/**  **Placebo** | **Risk of bias** | **Inconsistency** | **Indirectness** | **Imprecision** | **Publication bias** | **Quality of evidence** |
| --- | --- | --- | --- | --- | --- | --- | --- | --- | --- |
| **Effective rate** | 23 | 812/871 | 648/859 | some concerns | P =0.95; I² = 0% | No | RR 1.24 (1.18,1.29);  P＜0.00001 | No | ★★★☆  Moderate quality of evidence |
| **ALT (U/L)** | 27 | 920 | 906 | some concerns | P ＜0.00001; I² = 87% | No | MD -13.89 (-17.76,-10.02);  P＜0.00001 | No | ★★☆☆  Low quality of evidence |
| **AST (U/L)** | 11 | 362 | 359 | some concerns | P ＜0.00001; I² = 94% | No | MD -19.55 (-27.02,-12.08);  P＜0.00001 | No | ★★☆☆  Low quality of evidence |
| **GGT (U/L)** | 21 | 721 | 709 | some concerns | P ＜0.00001; I² = 98% | No | MD -30.82 (-42.60,-19.04);  P＜0.00001 | Probably yes | ★☆☆☆  Very low quality of evidence |
| **TBIL (μmol/L)** | 29 | 975 | 957 | some concerns | P＜0.00001; I² = 93% | No | MD 25.67 (-31.82,-19.52);  P＜0.00001 | No | ★★☆☆  Low quality of evidence |
| **DBIL (μmol/L)** | 23 | 759 | 746 | some concerns | P＜0.00001; I² = 97% | No | MD -20.27 (-26.15,-14.40);  P＜0.00001 | No | ★★☆☆  Low quality of evidence |
| **TBA (μmol/L)** | 18 | 606 | 593 | some concerns | P＜0.00001; I² = 93% | No | MD -25.68 (-31.33,-20.04);  P＜0.00001 | No | ★★☆☆  Low quality of evidence |
| **ADRs** | 17 | 83/630 | 72/631 | some concerns | P = 0. 15; I² = 0% | No | RD 0.03 (-0.01,0.06);  P =0.15 | No | ★★★☆  Moderate quality of evidence |
