# Supplementary material for: Efficacy and safety of ursodeoxycholic acid in children with cholestasis: A systematic review and meta-analysis
Source: PLoS One. 2023 Jan 31;18(1):e0280691. doi: 10.1371/journal.pone.0280691 (PMC9888709; doi:10.1371/journal.pone.0280691)
Supplement: S1 Fig — (PDF) [file pone.0280691.s002.pdf]

Study or Subgroup	Ursodeoxycholic acid Events	Total	Placebo/Blank control Events	Total	Weight	Risk Ratio M-H, Fixed, 95% CI
Brilliant 2016	41	43	34	43	5.2%	1.21 [1.02, 1.43]
Feng Lei 2017	53	55	43	55	6.6%	1.23 [1.06, 1.43]
Ge Xingjing 2014	18	20	16	20	2.5%	1.13 [0.86, 1.46]
High Venus 2009	42	45	31	40	5.0%	1.20 [1.00, 1.45]
Hu Haiyan 2020	32	35	28	35	4.3%	1.14 [0.94, 1.39]
Hu Huigang 2015	27	30	23	30	3.5%	1.17 [0.93, 1.48]
Hu Jinping 2013	32	32	25	33	3.8%	1.31 [1.08, 1.60]
Li Changxiao 2012	27	30	26	32	3.9%	1.11 [0.90, 1.36]
Lin Guidi 2019	33	35	27	35	4.1%	1.22 [1.00, 1.49]
Ma Qiushi 2019	34	36	28	36	4.3%	1.21 [1.00, 1.47]
Parhati Ablimiti 2016	26	30	21	30	3.2%	1.24 [0.94, 1.63]
Qin Zhen 2016	43	48	35	48	5.4%	1.23 [1.01, 1.50]
Sun Beibei 2014	24	26	14	20	2.4%	1.32 [0.97, 1.79]
Wang Wei 2018	49	51	41	51	6.3%	1.20 [1.03, 1.38]
Wu Bixia 2012	26	26	15	26	2.4%	1.71 [1.23, 2.38]
Wu Junfeng 2021	40	44	30	44	4.6%	1.33 [1.07, 1.67]
Wu Manpeng 2013	17	20	11	15	1.9%	1.16 [0.81, 1.66]
Xu Ya 2019	42	43	36	43	5.5%	1.17 [1.01, 1.34]
Yang Fengxia 2019	40	44	35	47	5.2%	1.22 [1.01, 1.48]
Yang Xingge 2019	57	64	48	64	7.4%	1.19 [1.01, 1.40]
Zhao Yang 2012	40	42	32	40	5.0%	1.19 [1.01, 1.41]
Zhong Qiong 2017	31	32	19	32	2.9%	1.63 [1.22, 2.19]
Zhou Li 2017	38	40	30	40	4.6%	1.27 [1.04, 1.54]
**Total (95% CI)**		**871**		**859**	**100.0%**	**1.24 [1.18, 1.29]**
Total events	812		648			

Heterogeneity: Chi² = 12.25, df = 22 (P = 0.95); I² = 0%

Test for overall effect: Z = 9.84 (P < 0.00001)

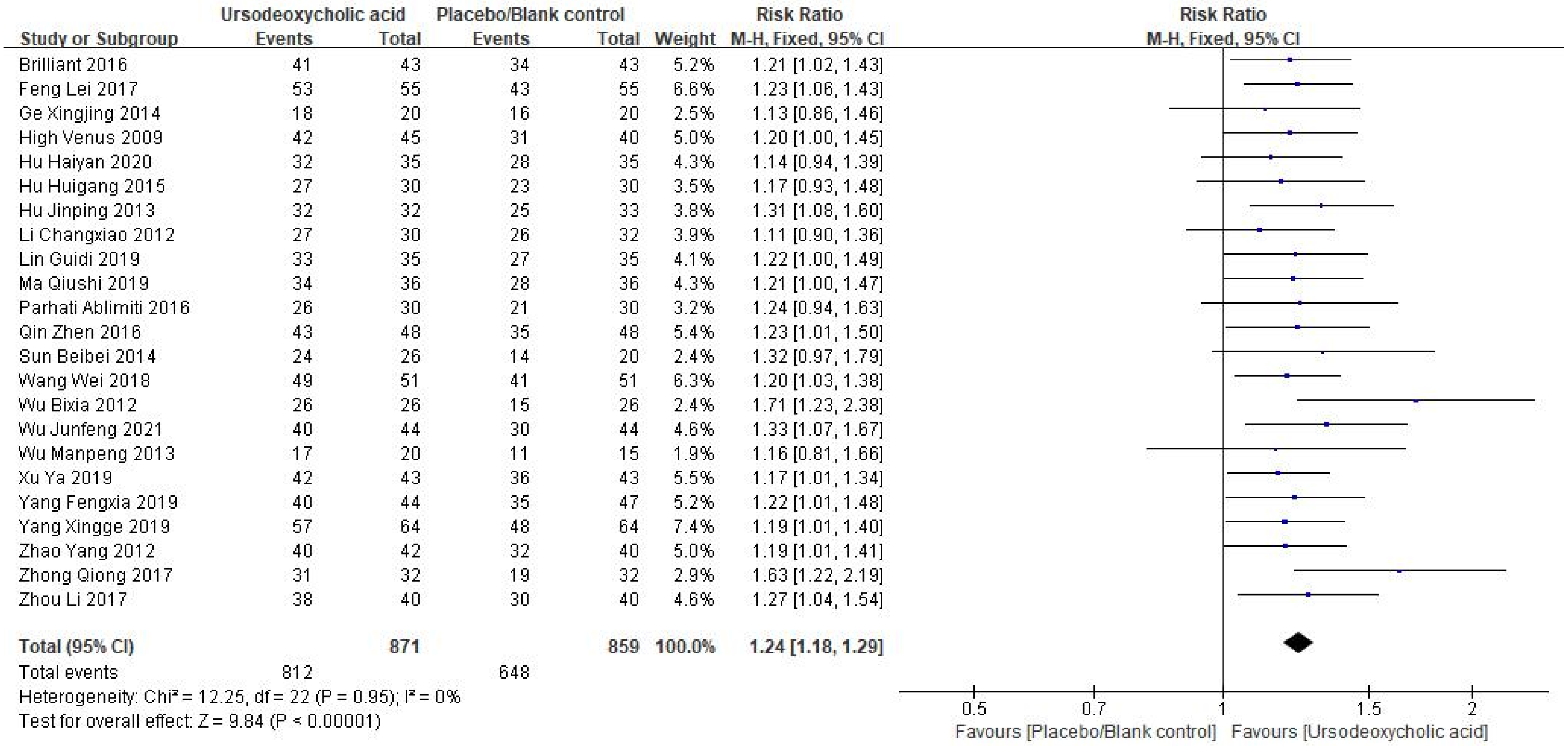

Figure 1 Meta-analysis forest plot of effective rate

Study or Subgroup	Ursodeoxycholic acid Mean	SD	Total	Placebo/Blank control Mean	SD	Total	Weight	Mean Difference IV, Random, 95% CI
Brilliant 2016	73.5	20.6	43	115.2	34.8	43	3.5%	-41.70 [-53.79, -29.61]
Fan Jianping 2008	68	21	29	124	48	25	2.8%	-56.00 [-76.31, -35.69]
Feng Lei 2017	73.52	20.37	55	115.61	36.58	55	3.6%	-42.09 [-53.16, -31.02]
Ge Xingjing 2014	30.2	15.8	20	41.8	17.4	20	3.6%	-11.60 [-21.90, -1.30]
High Venus 2009	89.33	27.46	45	122.68	12.57	40	3.7%	-33.35 [-42.27, -24.43]
Hu Haiyan 2020	151.33	31.32	35	128.42	24.63	35	3.4%	22.91 [9.71, 36.11]
Hu Huigang 2015	34.2	10.5	30	45.3	17.9	30	3.8%	-11.10 [-18.53, -3.67]
Hu Jinping 2013	28.6	15.8	32	43.6	18.5	33	3.8%	-15.00 [-23.36, -6.64]
Lan Jing 2022	155.6	7.41	34	181	11.2	34	3.9%	-25.40 [-29.91, -20.89]
Li Changxiao 2012	153.73	90.62	30	84.5	83.75	32	1.3%	69.23 [25.72, 112.74]
Lin Guidi 2019	46.87	9.55	35	74.23	12.54	35	3.9%	-27.36 [-32.58, -22.14]
Li Shumin 2013	64	15.3	20	81.4	13.5	20	3.7%	-17.40 [-26.34, -8.46]
Liu Yunfeng 2017	71.39	15.46	32	98.39	21.39	32	3.7%	-27.00 [-36.14, -17.86]
Ma Qiushi 2019	42.16	12.38	36	78.84	12.24	36	3.9%	-36.68 [-42.37, -30.99]
Parhati Ablimiti 2016	40.4	24.1	30	41	23.9	30	3.5%	-0.60 [-12.75, 11.55]
Qin Zhen 2016	44.3	9.27	48	77.31	18.04	48	3.9%	-33.01 [-38.75, -27.27]
Sun Beibei 2014	54.33	17.46	26	76.68	22.57	20	3.5%	-22.35 [-34.30, -10.40]
Tang Qing 2012	140.86	65.28	40	150.12	76.35	40	2.0%	-9.26 [-40.39, 21.87]
Wang Wei 2018	70.75	18.52	51	97.35	20.46	51	3.8%	-26.60 [-34.17, -19.03]
Wang Xiaoli 2012	67.2	5.8	12	78.5	6.3	10	3.9%	-11.30 [-16.40, -6.20]
Wang Yongbo 2009	33	8.7	13	61	12.6	13	3.8%	-28.00 [-36.32, -19.68]
Wu Bixia 2012	117.8	43.5	26	215.3	76.9	26	1.8%	-97.50 [-131.46, -63.54]
Wu Junfeng 2021	82.13	19.19	44	119.73	31.36	44	3.6%	-37.60 [-48.46, -26.74]
Wu Manpeng 2013	40.5	12.8	20	42.6	24.9	15	3.4%	-2.10 [-15.89, 11.69]
Xu Hong 2012	65.9	17.6	20	156.5	17.9	20	3.6%	-90.60 [-101.60, -79.60]
Xu Ya 2019	41.39	23.14	43	79.58	29.37	43	3.6%	-38.19 [-49.37, -27.01]
Yang Fengxia 2019	82.12	19.18	44	119.74	31.34	47	3.6%	-37.62 [-48.22, -27.02]
Zhao Yang 2012	28.7	16.7	42	42.7	19.3	40	3.8%	-14.00 [-21.83, -6.17]
Zhou Li 2017	68.9	11.4	40	85.6	22.3	40	3.8%	-16.70 [-24.46, -8.94]
**Total (95% CI)**			**975**			**957**	**100.0%**	**-25.67 [-31.82, -19.52]**

Heterogeneity: $Tau^2 = 244.43$; $Chi^2 = 387.99$, df = 28 (P < 0.00001); $I^2 = 93\%$

Test for overall effect: Z = 8.18 (P < 0.00001)

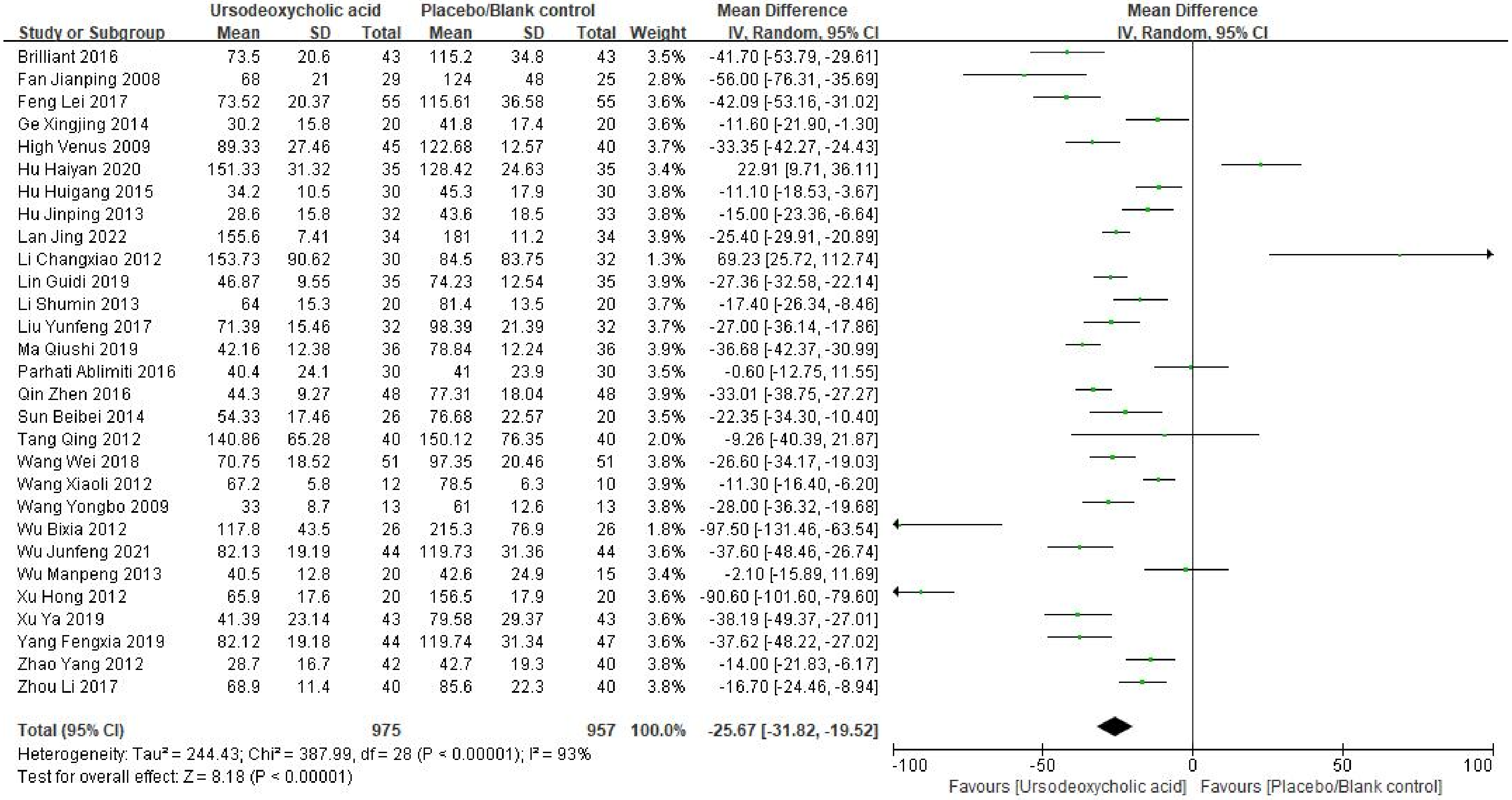

Figure 2 Meta-analysis forest plot of TBIL

Study or Subgroup	Ursodeoxycholic acid Mean	SD	Total	Placebo/Blank control Mean	SD	Total	Weight	Mean Difference IV, Random, 95% CI
Brilliant 2016	24.8	9.1	43	72.4	25.9	43	4.4%	-47.60 [-55.81, -39.39]
Fan Jianping 2008	25	17	29	62	28	25	4.0%	-37.00 [-49.60, -24.40]
Feng Lei 2017	34.87	9.11	55	72.44	25.63	55	4.5%	-37.57 [-44.76, -30.38]
Ge Xingjing 2014	15.7	10.9	20	25.7	13.5	20	4.5%	-10.00 [-17.60, -2.40]
High Venus 2009	39.59	15.39	45	58.9	7.28	40	4.7%	-19.31 [-24.34, -14.28]
Hu Haiyan 2020	59.32	17.95	35	87.56	18.57	35	4.4%	-28.24 [-36.80, -19.68]
Hu Huigang 2015	17.3	7.5	30	25.7	18.1	30	4.5%	-8.40 [-15.41, -1.39]
Hu Jinping 2013	16.5	9.4	32	24.7	11.2	33	4.7%	-8.20 [-13.22, -3.18]
Lan Jing 2022	36.42	3.61	34	47.51	4.14	34	4.8%	-11.09 [-12.94, -9.24]
Li Changxiao 2012	87.51	49.6	30	51.68	50.05	32	2.6%	35.83 [11.02, 60.64]
Lin Guidi 2019	53.39	8.83	35	37.44	5.67	35	4.8%	15.95 [12.47, 19.43]
Li Shumin 2013	35.4	12.6	20	53.8	15.2	20	4.4%	-18.40 [-27.05, -9.75]
Ma Qiushi 2019	32.18	12.41	36	62.37	11.62	36	4.7%	-30.19 [-35.74, -24.64]
Qin Zhen 2016	31.69	12.06	48	60.36	17.49	48	4.6%	-28.67 [-34.68, -22.66]
Sun Beibei 2014	29.39	14.39	26	50.9	17.28	20	4.3%	-21.51 [-30.89, -12.13]
Tang Qing 2012	78.64	32.57	40	88.65	44.37	40	3.4%	-10.01 [-27.07, 7.05]
Wang Xiaoli 2012	18.3	4.6	12	26.5	3.2	10	4.8%	-8.20 [-11.47, -4.93]
Wang Yongbo 2009	19	5.9	13	41	10.1	13	4.6%	-22.00 [-28.36, -15.64]
Wu Bixia 2012	52.6	30.7	26	145.8	65.4	26	2.3%	-93.20 [-120.97, -65.43]
Wu Junfeng 2021	35.81	10.06	44	70.07	17.61	44	4.6%	-34.26 [-40.25, -28.27]
Xu Hong 2012	10.5	3.2	20	27.4	4.3	20	4.8%	-16.90 [-19.25, -14.55]
Yang Fengxia 2019	35.82	10.05	44	70.09	17.63	47	4.6%	-34.27 [-40.12, -28.42]
Zhao Yang 2012	16.3	9.8	42	24.6	11.5	40	4.7%	-8.30 [-12.94, -3.66]
**Total (95% CI)**			**759**			**746**	**100.0%**	**-20.27 [-26.15, -14.40]**

Heterogeneity: $Tau^2 = 184.98$; $Chi^2 = 662.48$, df = 22 (P < 0.00001); $I^2 = 97\%$

Test for overall effect: Z = 6.76 (P < 0.00001)

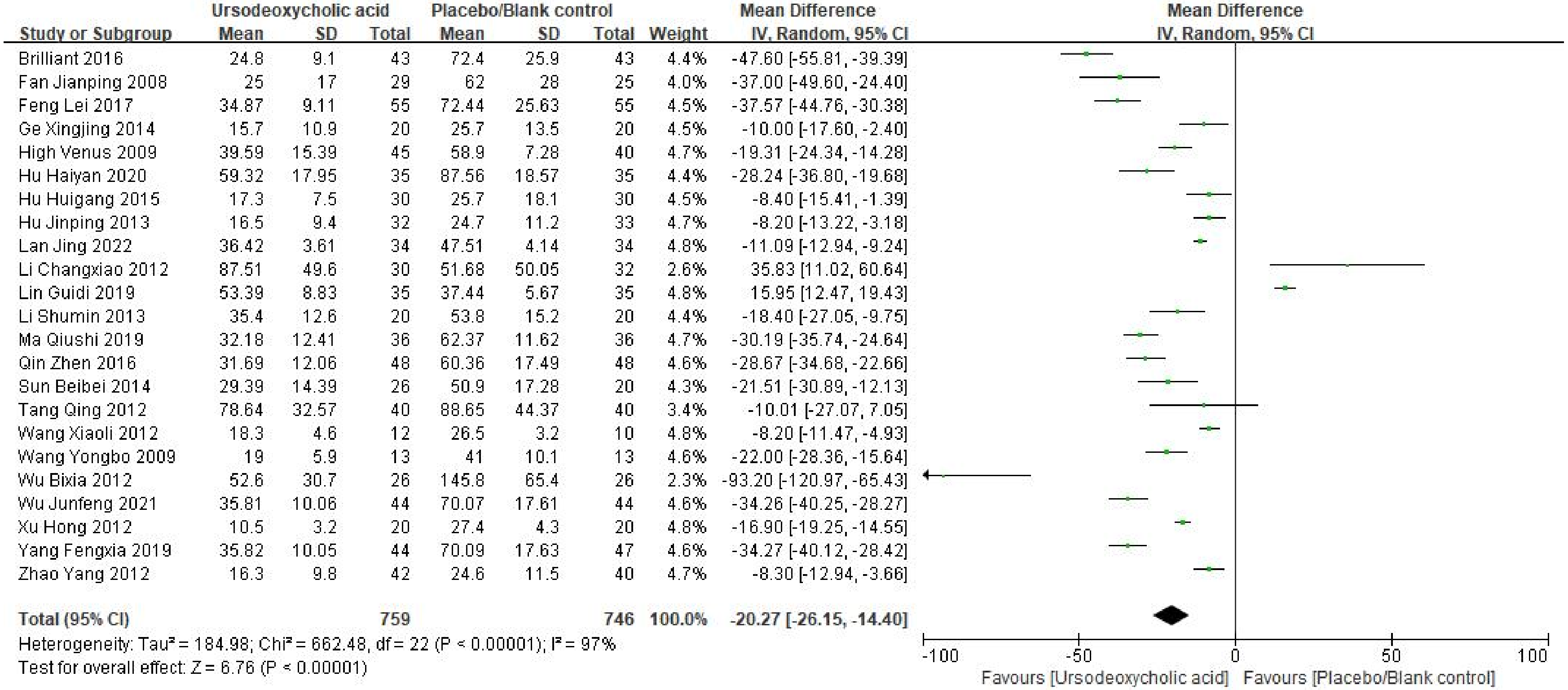

Figure 3 Meta-analysis forest plot of DBIL

Study or Subgroup	Ursodeoxycholic acid Mean	SD	Total	Placebo/Blank control Mean	SD	Total	Weight	Mean Difference IV, Random, 95% CI
Brilliant 2016	77.1	36.8	43	106.3	34.8	43	4.6%	-29.20 [-44.34, -14.06]
Fan Jianping 2008	28	20	29	68	25	25	5.3%	-40.00 [-52.21, -27.79]
Feng Lei 2017	77.15	36.84	55	105.87	35.25	55	5.0%	-28.72 [-42.20, -15.24]
Ge Xingjing 2014	30.7	15.8	20	40.9	28.3	20	4.8%	-10.20 [-24.40, 4.00]
High Venus 2009	68.25	18.69	45	98.29	19.85	40	6.1%	-30.04 [-38.27, -21.81]
Hu Huigang 2015	31.1	15.4	30	40.2	18.9	30	6.0%	-9.10 [-17.82, -0.38]
Hu Jinping 2013	31.5	11.6	32	52.3	27.4	33	5.7%	-20.80 [-30.98, -10.62]
Lan Jing 2022	30.28	3.52	34	50.88	5.8	34	6.9%	-20.60 [-22.88, -18.32]
Li Shumin 2013	43.7	9.2	20	96.7	14.6	20	6.2%	-53.00 [-60.56, -45.44]
Sun Beibei 2014	68.45	28.69	26	97.26	28.85	20	4.3%	-28.81 [-45.59, -12.03]
Wang Yongbo 2009	11.8	8.7	13	26.8	9.6	13	6.3%	-15.00 [-22.04, -7.96]
Wu Bixia 2012	23.7	12.8	26	62.8	24.5	26	5.6%	-39.10 [-49.73, -28.47]
Wu Junfeng 2021	30.26	5.44	44	40.64	6.51	44	6.9%	-10.38 [-12.89, -7.87]
Xu Hong 2012	31	10.9	20	53.2	18.9	20	5.8%	-22.20 [-31.76, -12.64]
Xu Ya 2019	78.51	33.29	43	108.62	34.61	43	4.8%	-30.11 [-44.46, -15.76]
Yang Fengxia 2019	30.27	5.45	44	40.69	6.53	47	6.9%	-10.42 [-12.89, -7.95]
Zhao Yang 2012	32.2	12.7	42	41.3	26.6	40	5.9%	-9.10 [-18.19, -0.01]
Zhou Li 2017	38.7	18.6	40	136.2	75.6	40	3.1%	-97.50 [-121.63, -73.37]
**Total (95% CI)**			**606**			**593**	**100.0%**	**-25.68 [-31.33, -20.04]**

Heterogeneity: Tau² = 119.05; Chi² = 252.81, df = 17 (P < 0.00001); I² = 93%
Test for overall effect: Z = 8.92 (P < 0.00001)

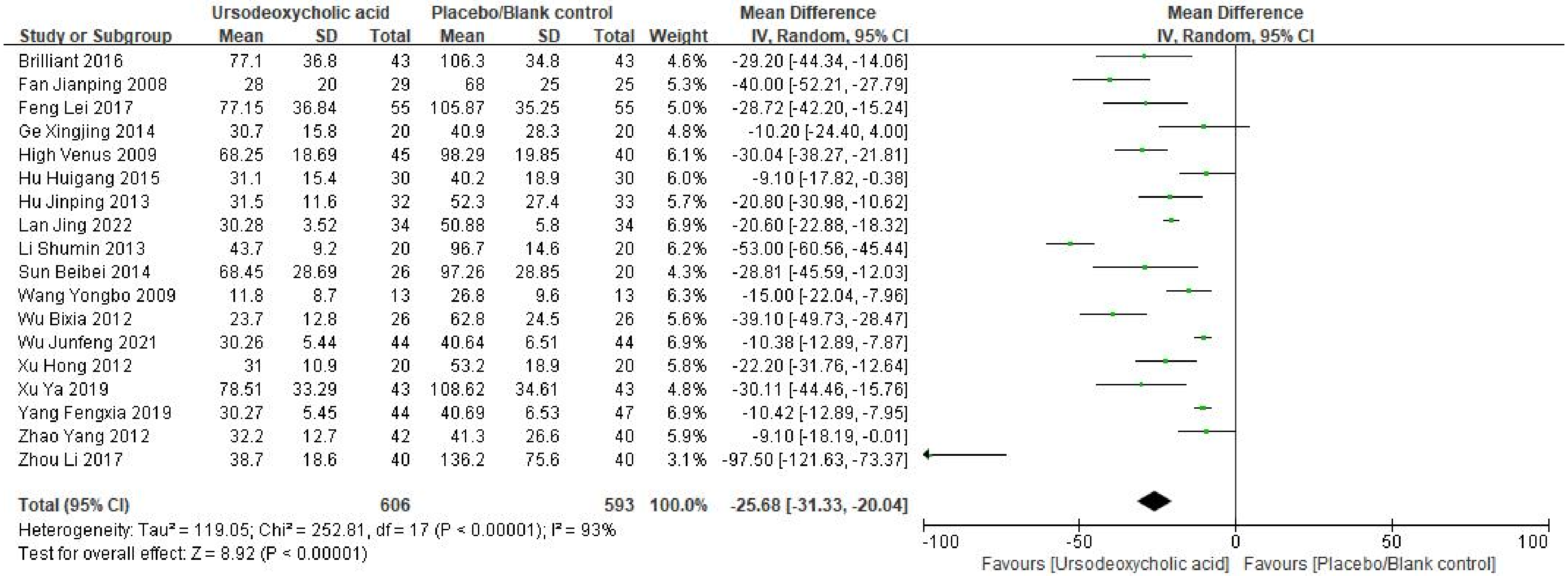

Figure 4 Meta-analysis forest plot of TBA

Study or Subgroup	Ursodeoxycholic acid Mean	SD	Total	Placebo/Blank control Mean	SD	Total	Weight	Mean Difference IV, Random, 95% CI
Brilliant 2016	62.1	13.8	43	79.1	23.6	43	4.0%	-17.00 [-25.17, -8.83]
Feng Lei 2017	62.35	13.1	55	78.18	24.1	55	4.2%	-15.83 [-23.08, -8.58]
Ge Xingjing 2014	40.7	20.5	20	50.3	21.6	20	3.2%	-9.60 [-22.65, 3.45]
High Venus 2009	60.46	8.28	45	93.5	9.76	40	4.7%	-33.04 [-36.91, -29.17]
Hu Haiyan 2020	125.31	16.22	35	133.52	16.48	35	4.1%	-8.21 [-15.87, -0.55]
Hu Huigang 2015	39.3	17.1	30	51.6	22.3	30	3.7%	-12.30 [-22.36, -2.24]
Hu Jinping 2013	32.1	17.5	32	54.8	23.2	33	3.7%	-22.70 [-32.67, -12.73]
Lan Jing 2022	26.08	4.33	34	36.85	6.19	34	4.8%	-10.77 [-13.31, -8.23]
Li Changxiao 2012	138.17	112.52	30	101.36	103.91	32	0.5%	36.81 [-17.20, 90.82]
Lin Guidi 2019	62.39	9.47	35	77.36	11.25	35	4.6%	-14.97 [-19.84, -10.10]
Li Shumin 2013	45.8	20.7	20	64.8	16.8	20	3.4%	-19.00 [-30.68, -7.32]
Liu Yunfeng 2017	42.73	10.79	32	59.43	15.52	32	4.3%	-16.70 [-23.25, -10.15]
Ma Qiushi 2019	87.25	14.37	36	97.48	14.62	36	4.3%	-10.23 [-16.93, -3.53]
Parhati Ablimiti 2016	50.2	10.3	30	60.9	38.7	30	2.9%	-10.70 [-25.03, 3.63]
Qin Zhen 2016	95.83	16.42	48	98.03	20.06	48	4.2%	-2.20 [-9.53, 5.13]
Sun Beibei 2014	96.05	29.76	26	62.46	18.28	20	3.0%	33.59 [19.62, 47.56]
Tang Qing 2012	77.35	51.26	40	82.58	63.42	40	1.6%	-5.23 [-30.50, 20.04]
Wang Wei 2018	41.18	9.36	51	57.83	11.57	51	4.7%	-16.65 [-20.73, -12.57]
Wang Xiaoli 2012	45.2	6.3	12	58.6	9.6	10	4.2%	-13.40 [-20.34, -6.46]
Wang Yongbo 2009	31	10.3	13	49	10.3	13	4.1%	-18.00 [-25.92, -10.08]
Wu Junfeng 2021	58.81	14.52	44	85.86	20.32	44	4.2%	-27.05 [-34.43, -19.67]
Wu Manpeng 2013	50.1	12.4	20	60.8	38.6	15	2.1%	-10.70 [-30.98, 9.58]
Xu Hong 2012	37.9	14.8	20	37.1	14.6	20	3.9%	0.80 [-8.31, 9.91]
Xu Ya 2019	50.37	15.69	43	76.18	38.24	43	3.3%	-25.81 [-38.16, -13.46]
Yang Fengxia 2019	58.8	14.51	44	85.87	20.33	47	4.2%	-27.07 [-34.29, -19.85]
Zhao Yang 2012	33.2	19.6	42	52.4	22.5	40	3.8%	-19.20 [-28.35, -10.05]
Zhou Li 2017	45.2	9.8	40	59.2	10.4	40	4.6%	-14.00 [-18.43, -9.57]
**Total (95% CI)**			**920**			**906**	**100.0%**	**-13.89 [-17.76, -10.02]**

Heterogeneity: Tau² = 79.66; Chi² = 203.13, df = 26 (P < 0.00001); I² = 87%

Test for overall effect: Z = 7.03 (P < 0.00001)

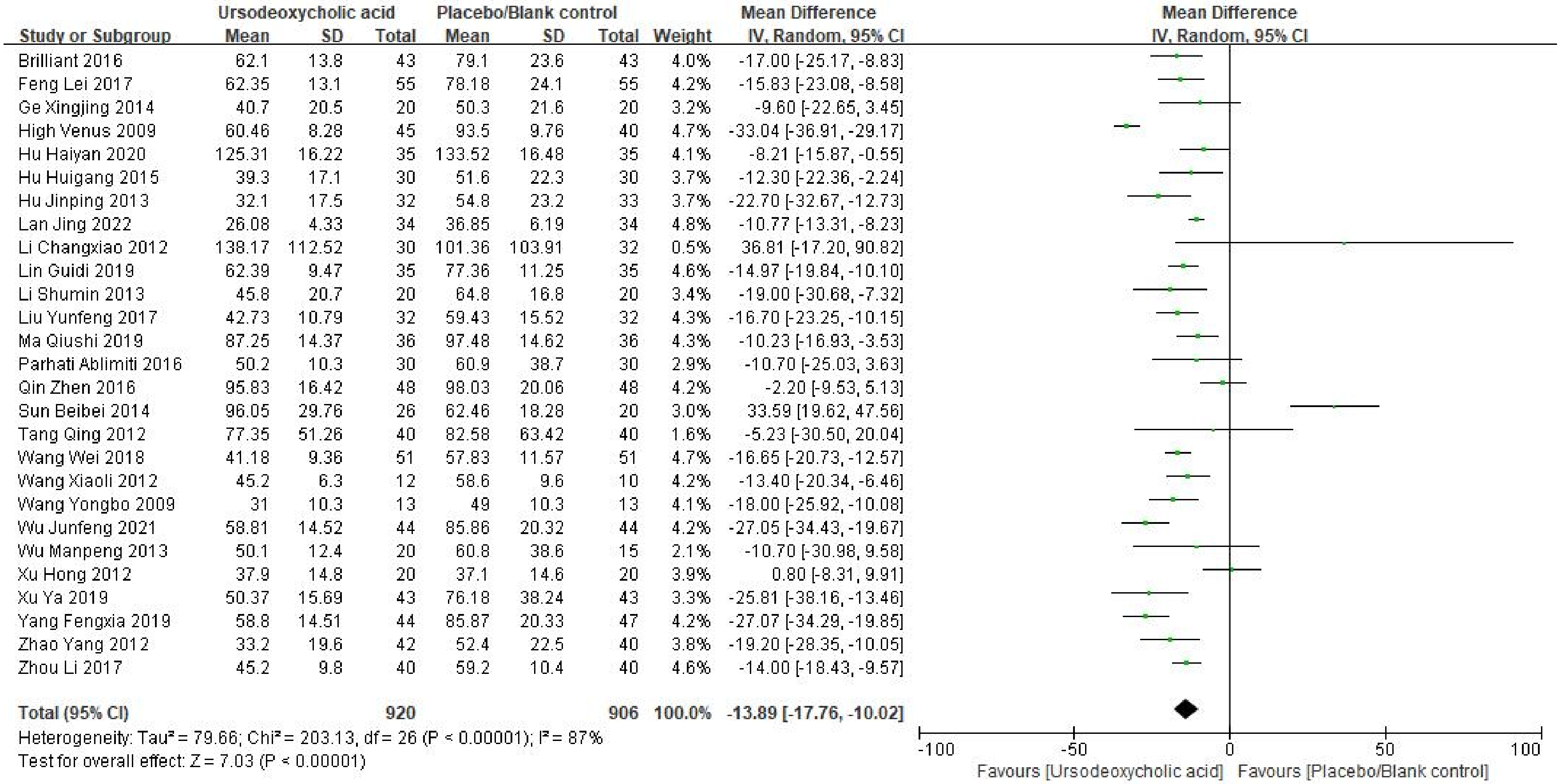

Figure 5 Meta-analysis forest plot of ALT

Study or Subgroup	Ursodeoxycholic acid Mean	SD	Total	Placebo/Blank control Mean	SD	Total	Weight	Mean Difference IV, Random, 95% CI
Hu Haiyan 2020	144.97	20.54	35	185.39	26.34	35	10.5%	-40.42 [-51.49, -29.35]
Lan Jing 2022	65.41	7.29	34	116.8	33.56	34	10.3%	-51.39 [-62.93, -39.85]
Li Changxiao 2012	211	197.71	30	131.12	105.48	32	0.8%	79.88 [0.25, 159.51]
Liu Yunfeng 2017	43.13	3.63	32	51.29	4.18	32	13.5%	-8.16 [-10.08, -6.24]
Ma Qiushi 2019	87.25	14.37	36	97.48	14.62	36	12.3%	-10.23 [-16.93, -3.53]
Parhati Ablimiti 2016	138.4	60.4	30	138.9	60.7	30	4.1%	-0.50 [-31.14, 30.14]
Qin Zhen 2016	123.07	12.27	48	128.63	19.02	48	12.4%	-5.56 [-11.96, 0.84]
Wang Wei 2018	43.36	4.59	51	50.52	5.16	51	13.5%	-7.16 [-9.06, -5.26]
Wu Bixia 2012	32.5	12.9	26	102.4	34.4	26	9.2%	-69.90 [-84.02, -55.78]
Wu Manpeng 2013	127.7	56.8	20	136.2	59.2	15	2.9%	-8.50 [-47.45, 30.45]
Xu Hong 2012	44.8	17.3	20	44.9	17.8	20	10.6%	-0.10 [-10.98, 10.78]
**Total (95% CI)**			**362**			**359**	**100.0%**	**-19.55 [-27.02, -12.08]**

Heterogeneity: Tau² = 106.55; Chi² = 167.46, df = 10 (P < 0.00001); I² = 94%
Test for overall effect: Z = 5.13 (P < 0.00001)

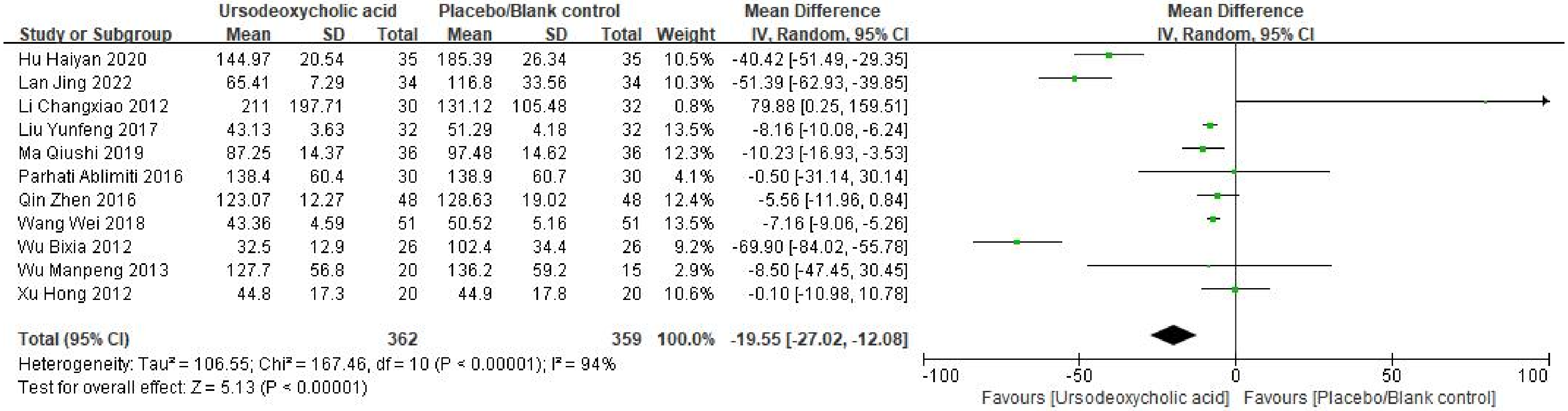

Figure 6 Meta-analysis forest plot of AST

Study or Subgroup	Ursodeoxycholic acid Mean	SD	Total	Placebo/Blank control Mean	SD	Total	Weight	Mean Difference IV, Random, 95% CI
Brilliant 2016	82.3	39.4	43	119.5	55.6	43	4.7%	-37.20 [-57.57, -16.83]
Fan Jianping 2008	102	37	29	52	22	25	5.0%	50.00 [34.01, 65.99]
Feng Lei 2017	82.15	38.67	55	118.67	56.81	55	4.9%	-36.52 [-54.68, -18.36]
Ge Xingjing 2014	80.7	45.1	20	94	46.3	20	4.2%	-13.30 [-41.63, 15.03]
High Venus 2009	109.36	24.27	45	155.4	12.92	40	5.4%	-46.04 [-54.18, -37.90]
Hu Huigang 2015	79.9	27.8	30	92.3	32.1	30	5.0%	-12.40 [-27.60, 2.80]
Hu Jinping 2013	73.6	35.2	32	98.7	46.3	33	4.8%	-25.10 [-45.06, -5.14]
Lan Jing 2022	215.6	19.38	34	300.8	27.34	34	5.2%	-85.20 [-96.46, -73.94]
Li Changxiao 2012	242.12	161.18	30	269.38	430.64	32	0.5%	-27.26 [-187.23, 132.71]
Lin Guidi 2019	39.47	6.22	35	51.23	9.13	35	5.5%	-11.76 [-15.42, -8.10]
Li Shumin 2013	63.5	18.5	20	95.6	21.5	20	5.2%	-32.10 [-44.53, -19.67]
Ma Qiushi 2019	125.57	19.46	36	177.56	20.42	36	5.3%	-51.99 [-61.20, -42.78]
Qin Zhen 2016	127.64	20.11	48	175.04	24.49	48	5.3%	-47.40 [-56.36, -38.44]
Sertac Arslanoglu 2007	56.1	36	15	71.9	29.1	14	4.5%	-15.80 [-39.55, 7.95]
Sun Beibei 2014	105.36	34.23	26	145.4	42.94	20	4.6%	-40.04 [-63.00, -17.08]
Tang Qing 2012	2.25	0.74	40	2.27	0.52	40	5.5%	-0.02 [-0.30, 0.26]
Wang Yongbo 2009	36.3	9.6	13	67.5	19.3	13	5.2%	-31.20 [-42.92, -19.48]
Wu Junfeng 2021	87.44	26.57	44	139.46	39.53	44	5.1%	-52.02 [-66.09, -37.95]
Yang Fengxia 2019	87.43	26.58	44	139.49	39.54	47	5.1%	-52.06 [-65.82, -38.30]
Zhao Yang 2012	84.3	47.9	42	98.4	45.2	40	4.7%	-14.10 [-34.25, 6.05]
Zhou Li 2017	90.5	51.3	40	150.6	68.5	40	4.3%	-60.10 [-86.62, -33.58]
**Total (95% CI)**			**721**			**709**	**100.0%**	**-30.82 [-42.60, -19.04]**

Heterogeneity: $Tau^2 = 656.39$; $Chi^2 = 875.14$, df = 20 ($P < 0.00001$); $I^2 = 98\%$
Test for overall effect: Z = 5.13 ($P < 0.00001$)

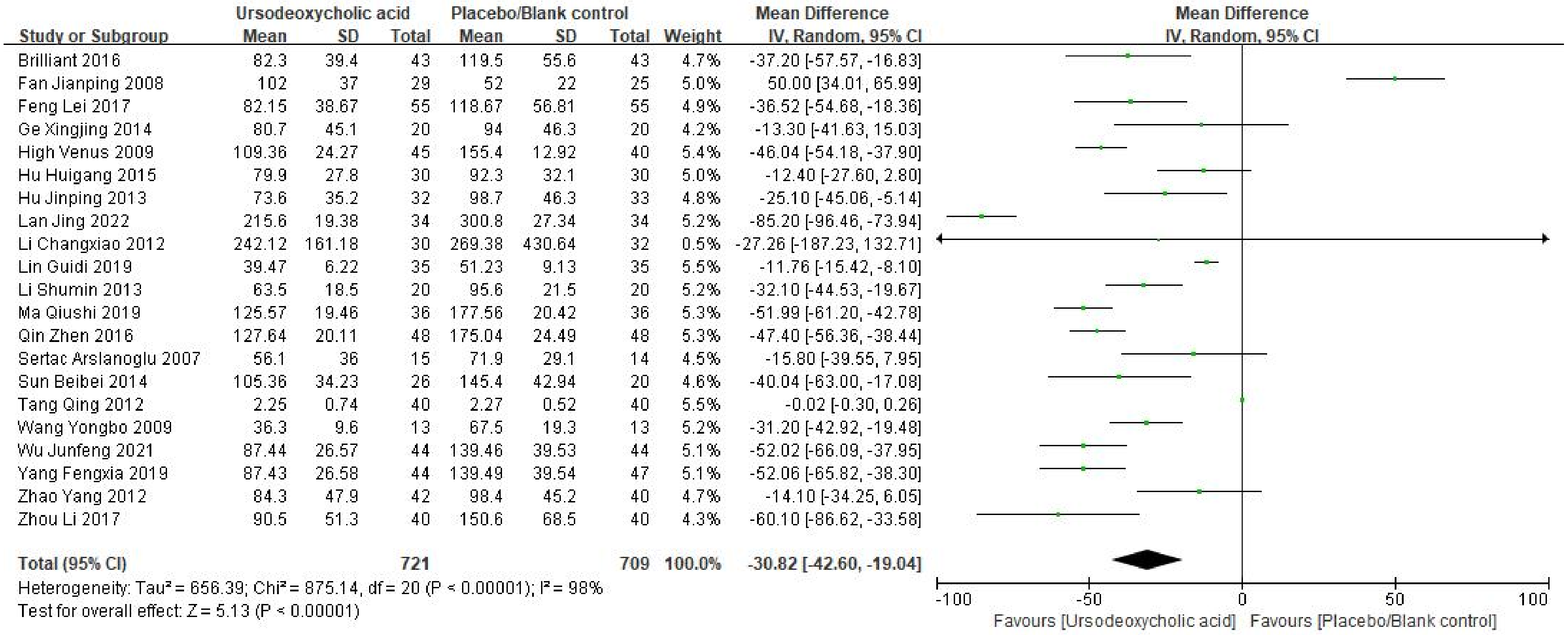

Figure 7 Meta-analysis forest plot of GGT

Study or Subgroup	Ursodeoxycholic acid Events	Ursodeoxycholic acid Total	Placebo/Blank control Events	Placebo/Blank control Total	Weight	Risk Difference M-H, Fixed, 95% CI
Brilliant 2016	0	43	0	43	6.8%	0.00 [-0.04, 0.04]
Ge Xingjing 2014	0	20	0	20	3.2%	0.00 [-0.09, 0.09]
Hu Haiyan 2020	5	35	8	35	5.6%	-0.09 [-0.27, 0.10]
Lan Jing 2022	19	34	12	43	6.0%	0.28 [0.07, 0.49]
Li Changxiao 2012	8	30	6	32	4.9%	0.08 [-0.13, 0.29]
Lin Guidi 2019	5	35	4	35	5.6%	0.03 [-0.13, 0.19]
Liu Yunfeng 2017	4	32	3	32	5.1%	0.03 [-0.12, 0.18]
Ma Qiushi 2019	5	36	5	36	5.7%	0.00 [-0.16, 0.16]
Qin Zhen 2016	4	48	3	48	7.6%	0.02 [-0.08, 0.12]
Sun Beibei 2014	0	26	0	20	3.6%	0.00 [-0.08, 0.08]
Wang Wei 2018	7	51	6	51	8.1%	0.02 [-0.11, 0.15]
Wu Bixia 2012	0	26	0	26	4.1%	0.00 [-0.07, 0.07]
Wu Junfeng 2021	6	44	9	44	7.0%	-0.07 [-0.22, 0.09]
Wu Manpeng 2013	0	20	0	15	2.7%	0.00 [-0.11, 0.11]
Yang Fengxia 2019	15	44	14	47	7.2%	0.04 [-0.15, 0.23]
Yang Xingge 2019	5	64	2	64	10.2%	0.05 [-0.03, 0.13]
Zhao Yang 2012	0	42	0	40	6.5%	0.00 [-0.05, 0.05]
**Total (95% CI)**		**630**		**631**	**100.0%**	**0.03 [-0.01, 0.06]**
Total events	83		72			

Heterogeneity: Chi² = 12.72, df = 16 (P = 0.69); I² = 0%
Test for overall effect: Z = 1.44 (P = 0.15)

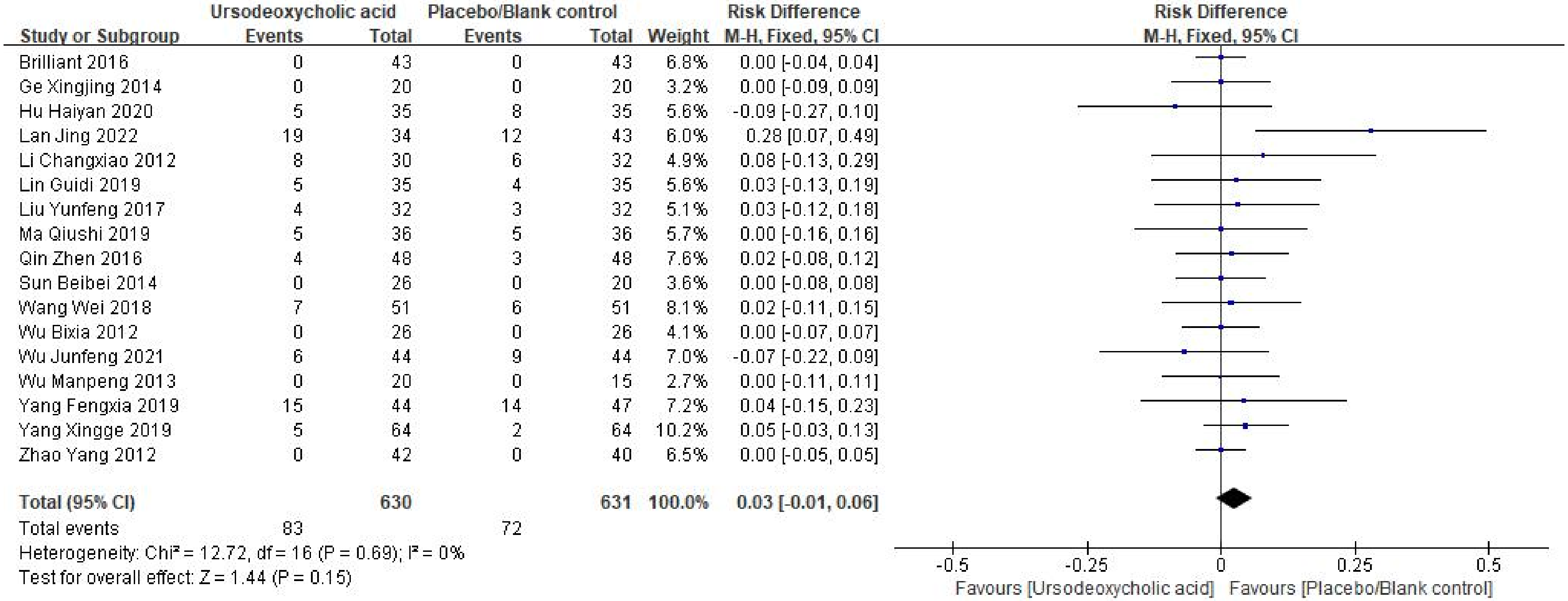

Figure 8 Meta-analysis forest plot of ADRs
