# Supplementary figures and images for: Efficacy and safety of ursodeoxycholic acid in children with cholestasis: A systematic review and meta-analysis
Source: PLoS One. 2023 Jan 31;18(1):e0280691. doi: 10.1371/journal.pone.0280691 (PMC9888709; doi:10.1371/journal.pone.0280691)

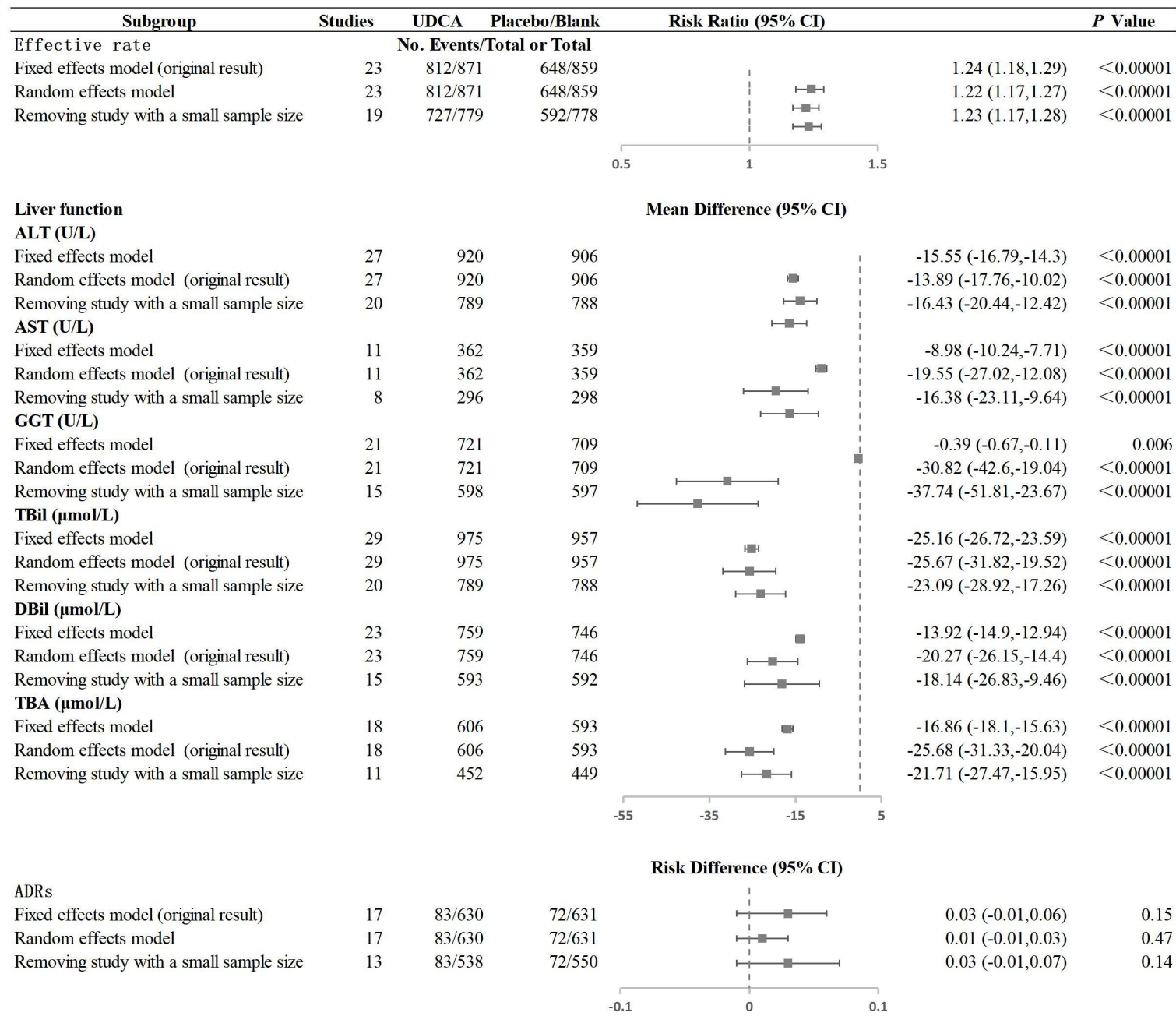

Figure Results of sensitivity analyses

Supplement: S2 Fig — (PDF) [file pone.0280691.s003.pdf]
