# Supplementary material for: Efficacy and safety of ursodeoxycholic acid in children with cholestasis: A systematic review and meta-analysis
Source: PLoS One. 2023 Jan 31;18(1):e0280691. doi: 10.1371/journal.pone.0280691 (PMC9888709; doi:10.1371/journal.pone.0280691)
Supplement: S3 Fig — (PDF) [file pone.0280691.s004.pdf]

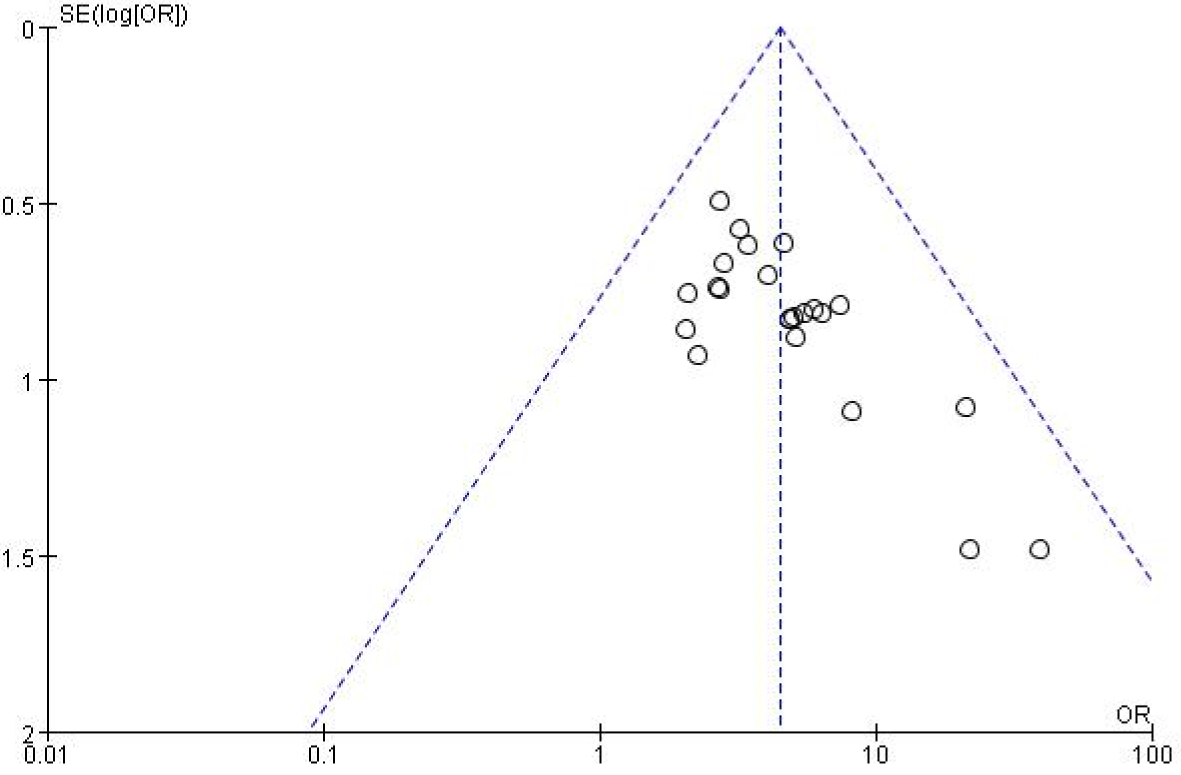

Figure 1 Funnel plot of effect rate

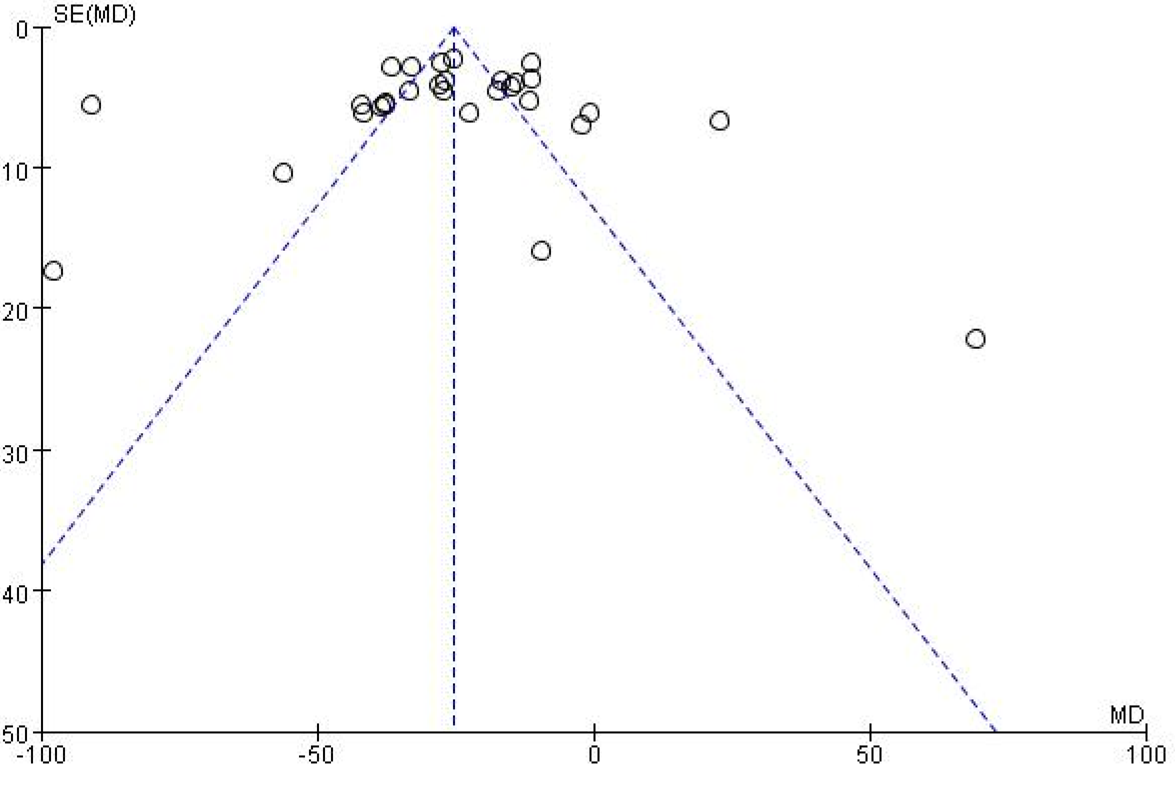

Figure 2 Funnel plot of TBIL

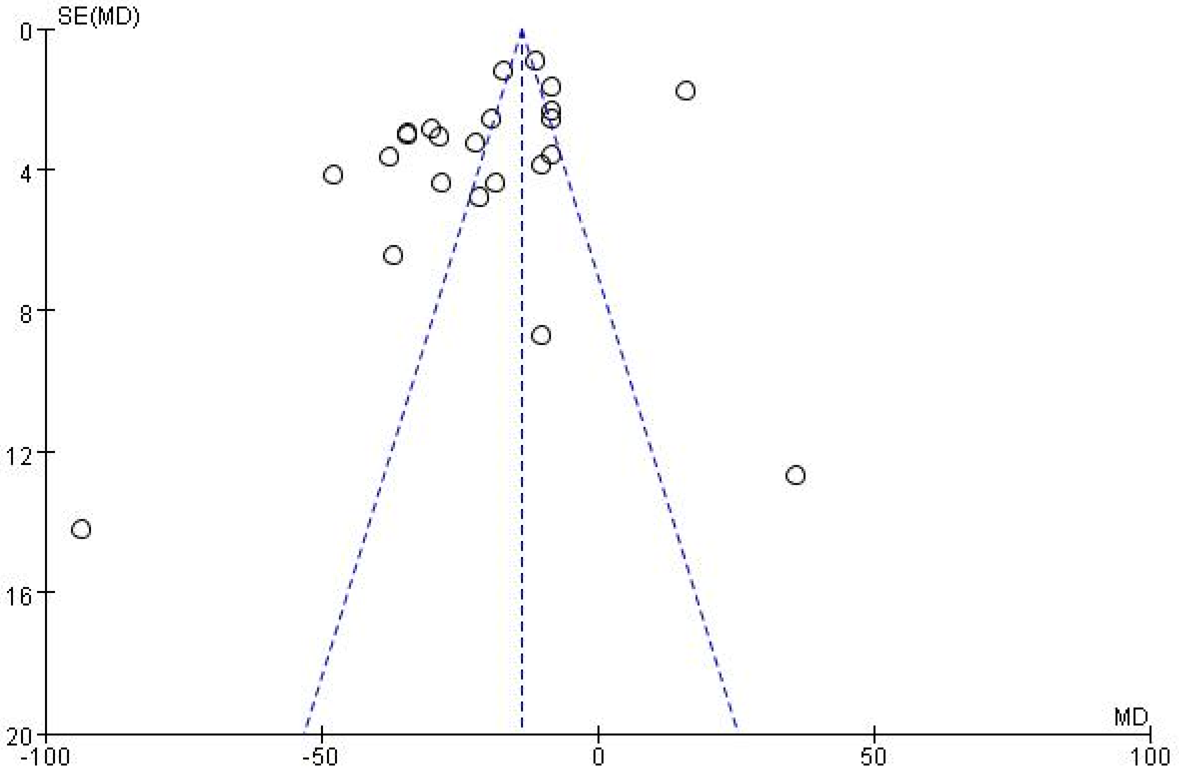

Figure 3 Funnel plot of TDIL

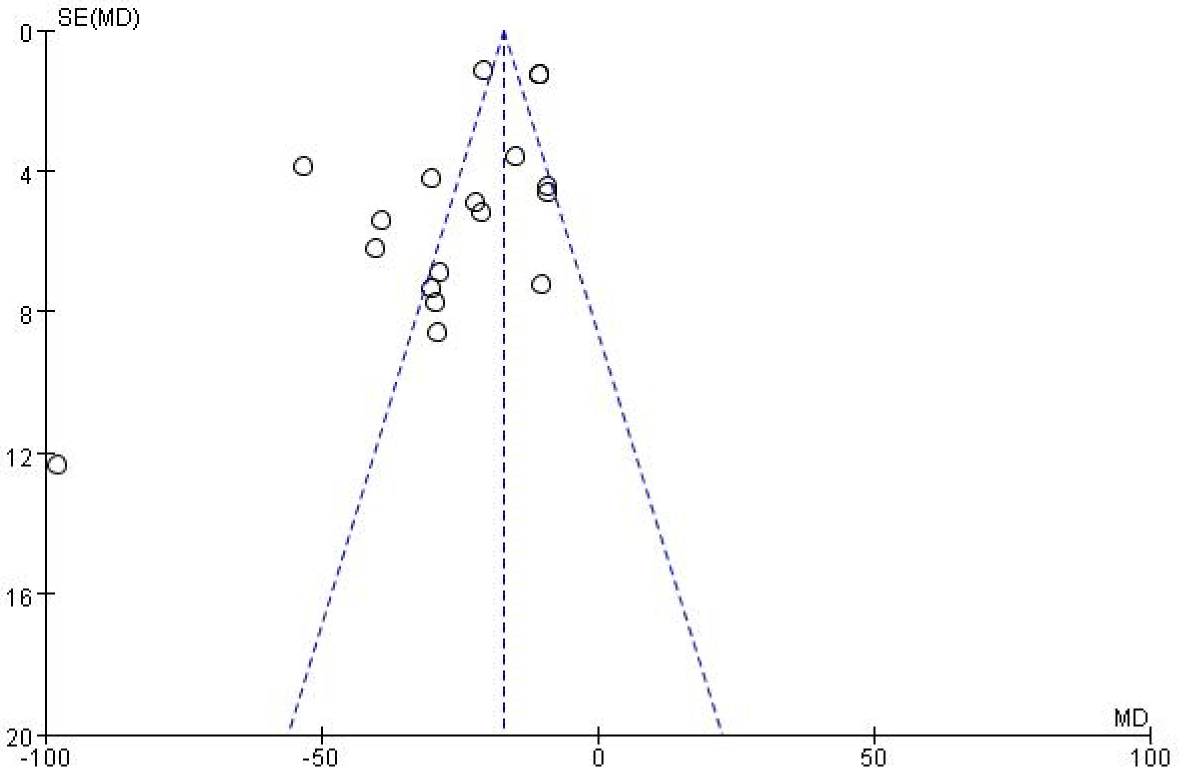

Figure 4 Funnel plot of TBA

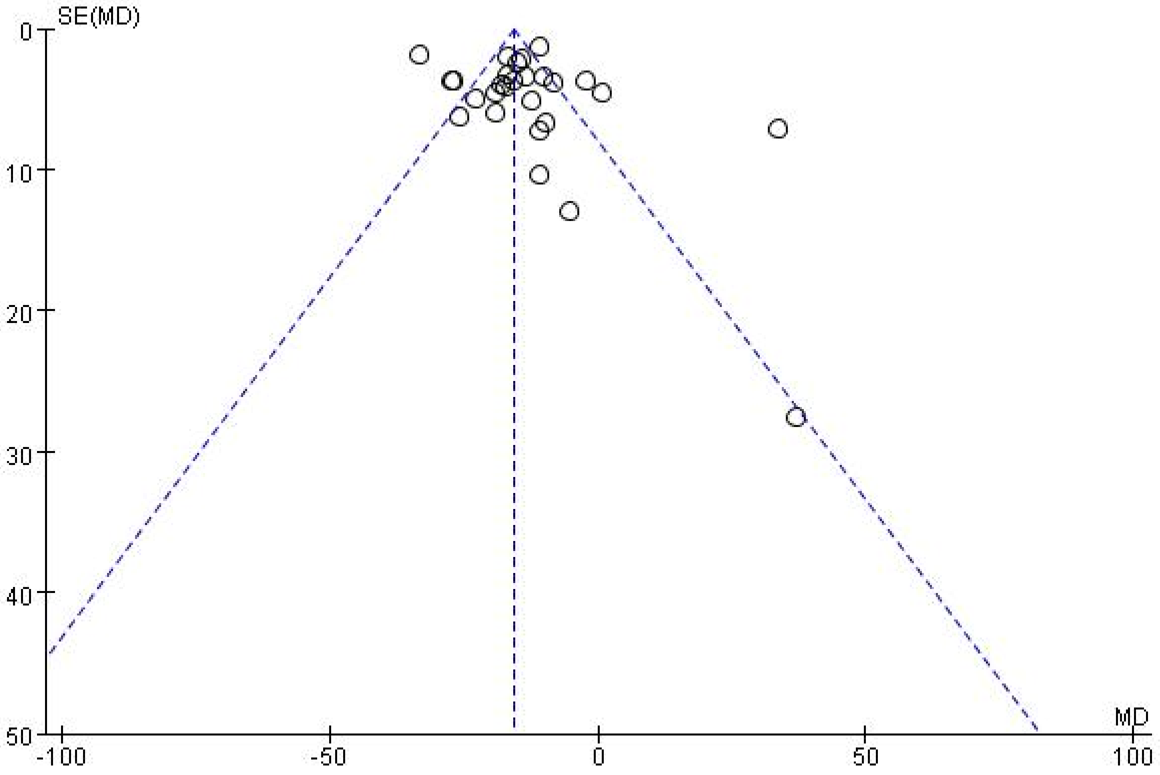

Figure 5 Funnel plot of ALT

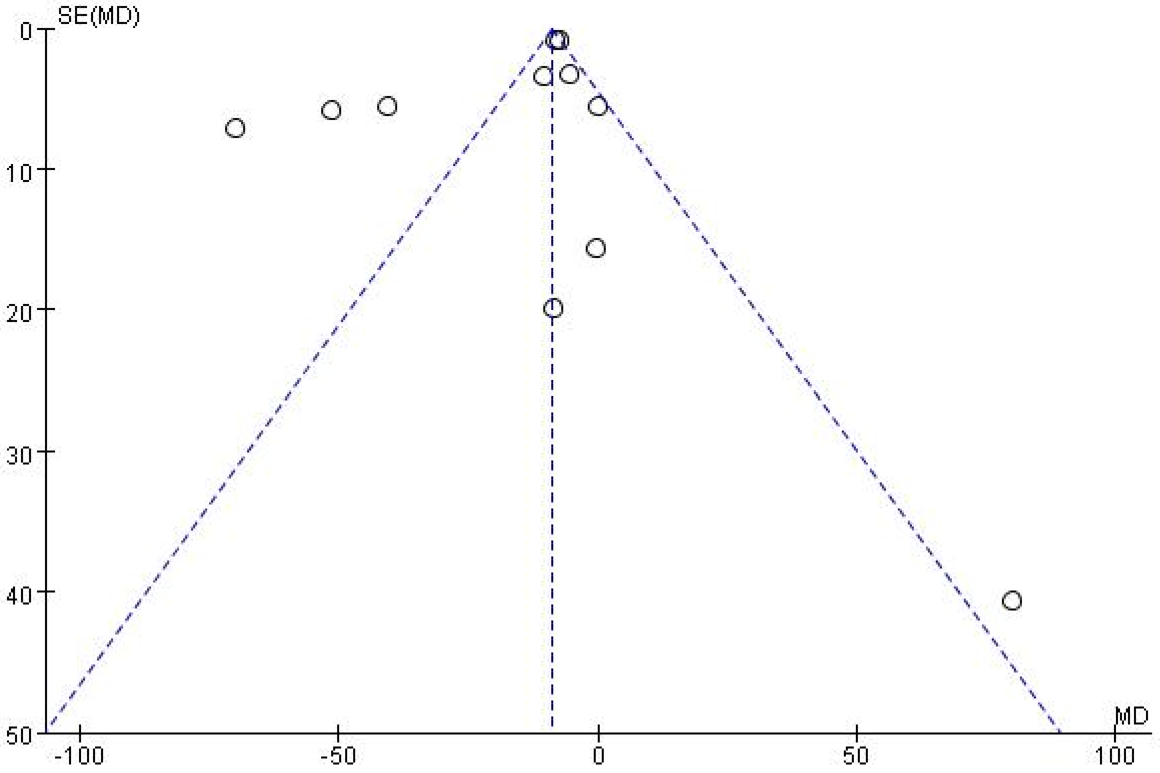

Figure 6 Funnel plot of AST

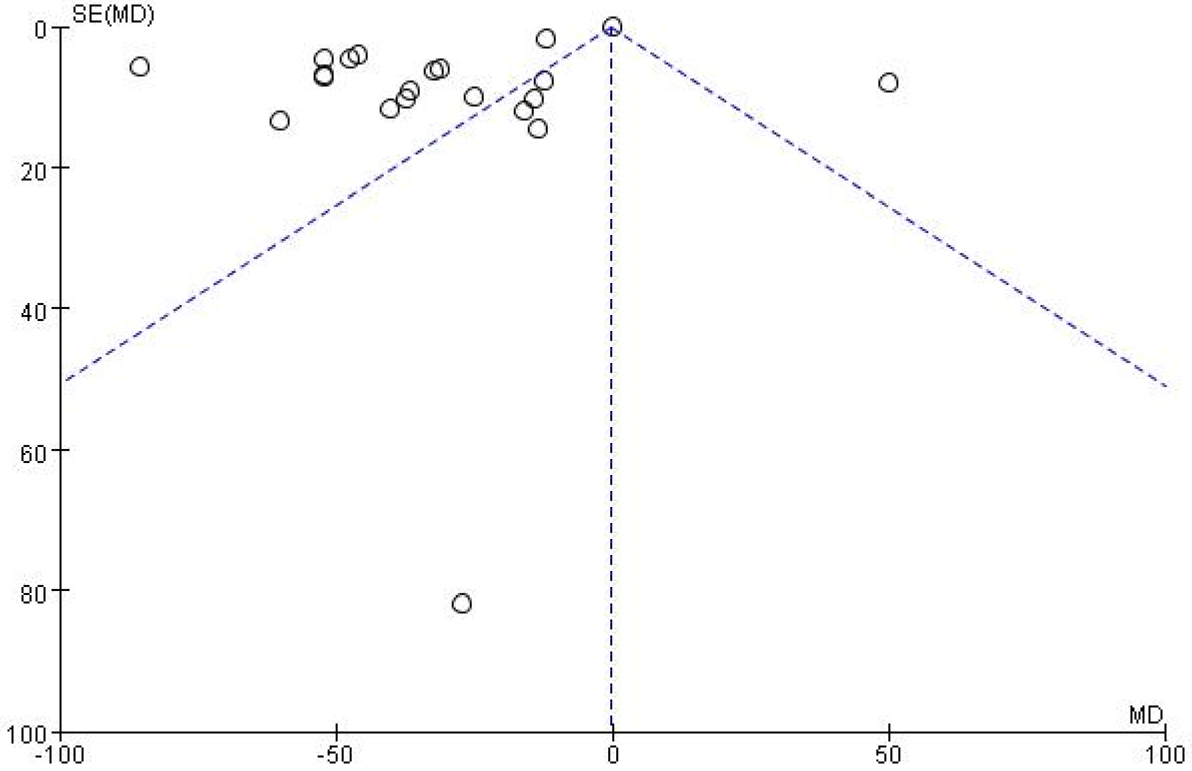

Figure 7 Funnel plot of GGT

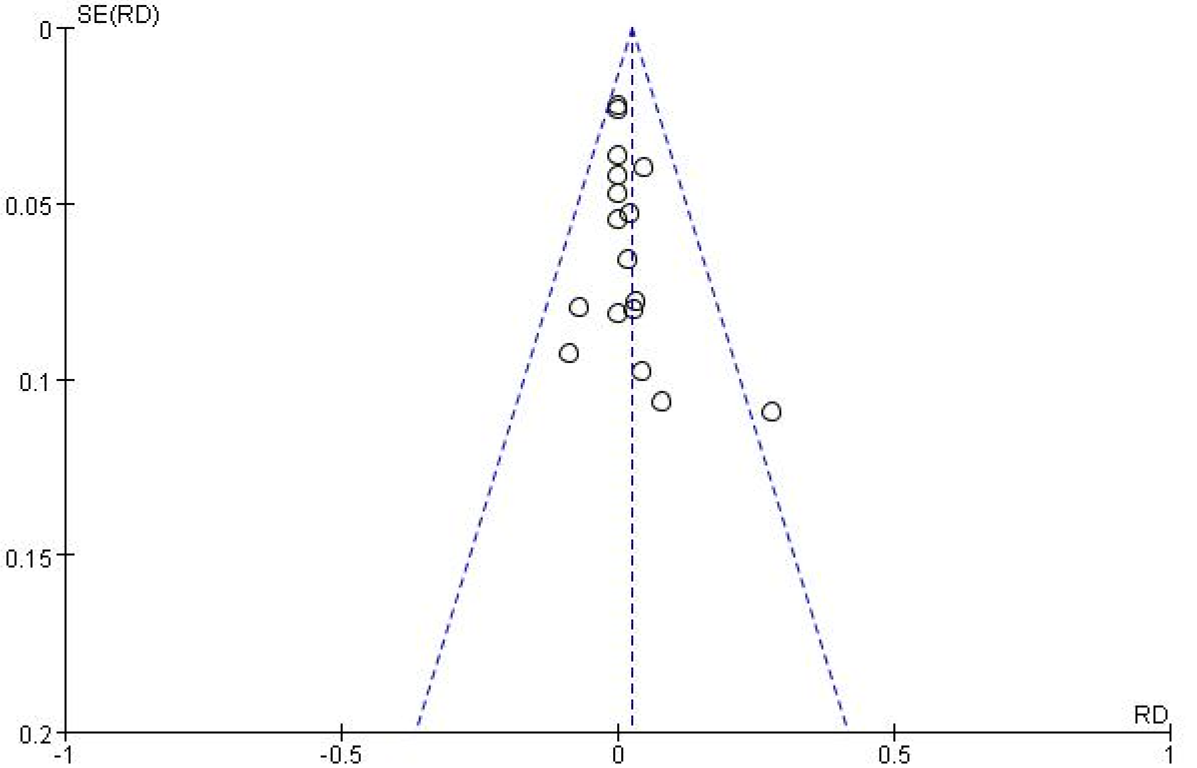

Figure 8 Funnel plot of ADRs
